# *Lrh1* can help reprogram sexual cell fate and is required for Sertoli cell development and spermatogenesis in the mouse testis

**DOI:** 10.1371/journal.pgen.1010088

**Published:** 2022-02-22

**Authors:** Kellie S. Agrimson, Anna Minkina, Danielle Sadowski, Andrew Wheeler, Mark W. Murphy, Micah D. Gearhart, Vivian J. Bardwell, David Zarkower

**Affiliations:** 1 Developmental Biology Center and Department of Genetics, Cell Biology, and Development, University of Minnesota, Minneapolis, Minnesota, United States of America; 2 University of Minnesota Masonic Cancer Center, Minneapolis, Minnesota, United States of America; University of Pennsylvania, UNITED STATES

## Abstract

The mammalian nuclear hormone receptors LRH1 (NR5A2) and SF1 (NR5A1) are close paralogs that can bind the same DNA motif and play crucial roles in gonadal development and function. *Lrh1* is essential for follicle development in the ovary and has been proposed to regulate steroidogenesis in the testis. *Lrh1* expression in the testis is highly elevated by loss of the sex regulator *Dmrt1*, which triggers male-to-female transdifferentiation of Sertoli cells. While *Sf1* has a well-defined and crucial role in testis development, no function for *Lrh1* in the male gonad has been reported. Here we use conditional genetics to examine *Lrh1* requirements both in gonadal cell fate reprogramming and in normal development of the three major cell lineages of the mouse testis. We find that loss of *Lrh1* suppresses sexual transdifferentiation, confirming that *Lrh1* can act as a key driver in reprogramming sexual cell fate. In otherwise wild-type testes, we find that *Lrh1* is dispensable in Leydig cells but is required in Sertoli cells for their proliferation, for seminiferous tubule morphogenesis, for maintenance of the blood-testis barrier, for feedback regulation of androgen production, and for support of spermatogenesis. Expression profiling identified misexpressed genes likely underlying most aspects of the Sertoli cell phenotype. In the germ line we found that *Lrh1* is required for maintenance of functional spermatogonia, and hence mutants progressively lose spermatogenesis. Reduced expression of the RNA binding factor *Nxf2* likely contributes to the SSC defect. Unexpectedly, however, over time the *Lrh1* mutant germ line recovered abundant spermatogenesis and fertility. This finding indicates that severe germ line depletion triggers a response allowing mutant spermatogonia to recover the ability to undergo complete spermatogenesis. Our results demonstrate that *Lrh1*, like *Sf1*, is an essential regulator of testis development and function but has a very distinct repertoire of functions.

## Author summary

Liver receptor homolog 1 (*Lrh1*) and its sister gene steroidogenic factor 1 (*Sf1*) are implicated in a number of aspects of gonadal development and function in mammals. *Sf1* plays essential roles in the gonads of both sexes; *Lrh1* is important in the ovary but its role in the testis has not been reported. We therefore used mouse genetics to ask whether *Lrh1* has any requirements in the testis. We found that *Lrh1* is essential for establishment and function of Sertoli cells, which provide support for germ cell development. We also found that *Lrh1* is required in the germ cells themselves to maintain functional stem cells that allow males to produce vast quantities of sperm throughout adult life. Surprisingly, after spermatogenesis failed in mutant males it recovered, and the mutant sperm were capable of fertilization. This finding demonstrates that severe germ cell depletion can trigger a response allowing spermatogenesis to occur even in the absence of *Lrh1*. We also found that *Lrh1* is required for male-to-female reprogramming of gonadal cells. Together our results establish that *Lrh1*, like *Sf1*, is a key regulator of testis development and function, but with distinct functions. Our results also extend the potential use of LRH1 in reprogramming cell types.

## Introduction

Formation of the mammalian gonad requires the coalescence of migrating primordial germ cells with somatic progenitor cells during fetal development, followed by complex and sexually dimorphic growth and differentiation programs that give rise to either male testes or female ovaries [1]. In both sexes the gonads play dual roles: they produce sex hormones and other signaling molecules that direct and support sexual differentiation and reproduction; and they produce gametes, either sperm or oocytes, that perpetuate the genome. The gonads become morphologically distinct and produce sexually specialized signaling molecules by about 13.5 days of embryonic development (E13.5). Gametogenesis begins shortly after birth in both sexes, and in males it continues life-long, supported by a robust and renewable population of spermatogonial stem cells (SSCs) [2].

Somatic sexual fate and the SSC population normally are stable but both require long-term maintenance. In the testis and the ovary, loss of key cell fate regulators (for example *Dmrt1* in males or *Foxl2* in females) can cause somatic cells to undergo sexual cell fate transdifferentiation, even in fully differentiated adult gonads [3–5]. The result is a reprogramming of male Sertoli cells to female granulosa-like cells or vice versa. This surprising finding demonstrates that continuous maintenance of somatic sex is required in the postnatal gonads of both sexes. In the germ line, loss of SSC regulators can result in a gradual depletion of SSCs, and hence of spermatogenesis, due to reduced self-renewal, proliferation, or both [6–8].

Among the major regulators of gonadal differentiation and function are a number of members of the nuclear hormone receptor transcription family. Two closely related family members, Steroidogenic factor 1 (*Sf1*; *Nr5a1*) and Liver receptor homolog 1 (*Lrh1; Nr5a2*) play crucial roles in the mammalian gonad [9]. These two genes formed by duplication in an ancestral vertebrate [10] and encode transcription factors that bind to similar or identical DNA motifs [11] (jaspar.genereg.net). Despite their identical or near-identical DNA binding properties [11–13], interaction with shared coregulators [9,14,15], and expression in an overlapping set of tissues and organs [9,16], SF1 and LRH1 have a number of distinct functions. These differences likely derive in part from differential expression levels in different cell types and in part from distinct coregulator interactions [9].

*Sf1* and *Lrh1* perform multiple roles in the gonads. One of these is steroidogenesis, and the two regulators are proposed to control key enzymes in steroidogenic cells both in the testis (in Leydig and Sertoli cells) and the ovary (in theca and granulosa cells) [9]. *Sf1* expression has been reported to be higher in Sertoli and theca cells and *Lrh1* higher in Leydig and granulosa cells [17–20]. *Lrh1* expression has also been reported in meiotic and postmeiotic germ cells in the rat testis [17]. In the mouse testis *Sf1* also promotes Mullerian duct regression by activating anti-Mullerian hormone (AMH) expression in Sertoli cells, regulates Leydig cell formation and survival, and promotes Sertoli cell differentiation and survival [21–23]. Indeed, heterozygosity of human *SF1* is a major cause of 46,XY gonadal dysgenesis (a difference in sex development, or DSD), resulting in male-to-female sex reversal [24,25]. In females, deletion of *Sf1* in granulosa cells in mice causes hypoplastic ovaries and infertility [26]. *Lrh1* also is important in the ovary. Homozygous null mutations in *Lrh1* are lethal due to embryonic arrest [27,28] but heterozygous females are viable and have impaired luteal function and reduced fertility [29]. Conditional deletion of *Lrh1* in granulosa cells affects activation of primordial follicles and granulosa cell proliferation and function, and the conditionally mutant females are anovulatory and infertile [19,20,30,31]. In the testis, despite reports of expression in Leydig, Sertoli, and germ cells [17,32], the function of *Lrh1* is poorly understood. Heterozygosity of *Lrh1* in the mouse caused reduced circulating testosterone levels [33], either via a direct role in testicular steroidogenesis or an indirect effect through regulation of the hypothalamic-pituitary-gonadal axis.

LRH1 and SF1 also can regulate stem cell pluripotency and differentiation: either protein can replace OCT4 in embryonic stem cells and either can induce differentiation of mesenchymal stem cells [34,35]. Moreover, LRH1 can replace OCT4 in generation of induced pluripotent stem cells (iPSCs) [36]. Thus SF1 and LRH1 can function either to promote pluripotency or to promote differentiation, depending on cellular context.

This study focuses on the role of *Lrh1* in the mouse testis. In wild-type animals *Lrh1* is expressed at much higher levels in the ovary than the testis, consistent with its well established roles in granulosa cells. In XY gonads mutant for *Dmrt1*, however, we previously found that *Lrh1* is highly upregulated prior to and during male-to-female transdifferentiation, consistent with it potentially playing a role in reprogramming Sertoli cells to granulosa-like cells. We have exploited a conditional *Lrh1* allele [37] to test the role of *Lrh1* in this transdifferentiation as well as to ask whether *Lrh1*, like *Sf1*, is important for normal development and function of the testis.

Using the conditional allele we find that *Lrh1* plays a crucial role in sexual cell fate reprogramming: loss of *Lrh1* in Sertoli cells largely blocked male-to-female transdifferentiation in *Dmrt1* conditional mutant testes. Despite reports that *Lrh1* is important for male steroidogenesis and is expressed at higher levels in Leydig than Sertoli cells, analysis of recent transcriptome data suggests it is not expressed in these cells, and we find that deletion of *Lrh1* in Leydig cells has no obvious effect on testicular morphology or fertility. However, transcriptome data indicate that *Lrh1* is expressed in neonatal and juvenile Sertoli cells. We find that *Lrh1* is required for normal neonatal Sertoli cell expansion and for perinatal morphogenesis of seminiferous tubules, with mutant tubules disordered and deficient in blood-testis barrier function and unable to properly support spermatogenesis. *Lrh1* also is expressed in undifferentiated spermatogonia including SSCs, and germ cell-specific loss of *Lrh1* caused progressive loss of spermatogenesis, consistent with a failure of SSC maintenance and/or self-renewal. Unexpectedly, we found that *Lrh1* mutant germ cells can regenerate to restore full spermatogenesis and can contribute to functional spermatozoa that pass the mutant allele to offspring. This regeneration occurs later in *Lrh1* heterozygous germlines, which undergo a slower depletion. Our results indicate that *Lrh1* activity is crucial for normal SSC maintenance or differentiation but mutant cells can be activated to restore fertility after germ cell depletion crosses a threshold level.

## Results

### *Lrh1* is expressed in juvenile Sertoli cells and in putative SSCs in the mouse testis

We first examined *Lrh1* expression to help gauge which cell types might require *Lrh1* function. We were unable to identify a specific antibody for LRH1 immunofluorescence or an efficient in situ hybridization probe against the region deleted by the *Lrh1* conditional allele. We therefore examined published mRNA sequencing data, comparing expression of *Lrh1* to that of *Sf1* and key cell type-specific markers. In transcriptome data from purified populations of fetal pre-Sertoli and postnatal Sertoli cells, *Lrh1* mRNA was not detectable between E11.5 and E13.5 [38], but it was present at low levels relative to *Sf1* in juvenile Sertoli cells at P5, P7 and P10 [39,40] and then it was very low or absent in P18 and adult Sertoli cells [39]. In fetal and adult Leydig cells (E14.5, E18.5, P10, P21, P56), *Sf1* mRNA was abundant as expected but, counter to some previous reports, *Lrh1* expression was not detected in Leydig cells at any stage [41]. Thus *Lrh1* appears to be expressed in juvenile Sertoli cells but not in Leydig cells.

**Fig 1:**
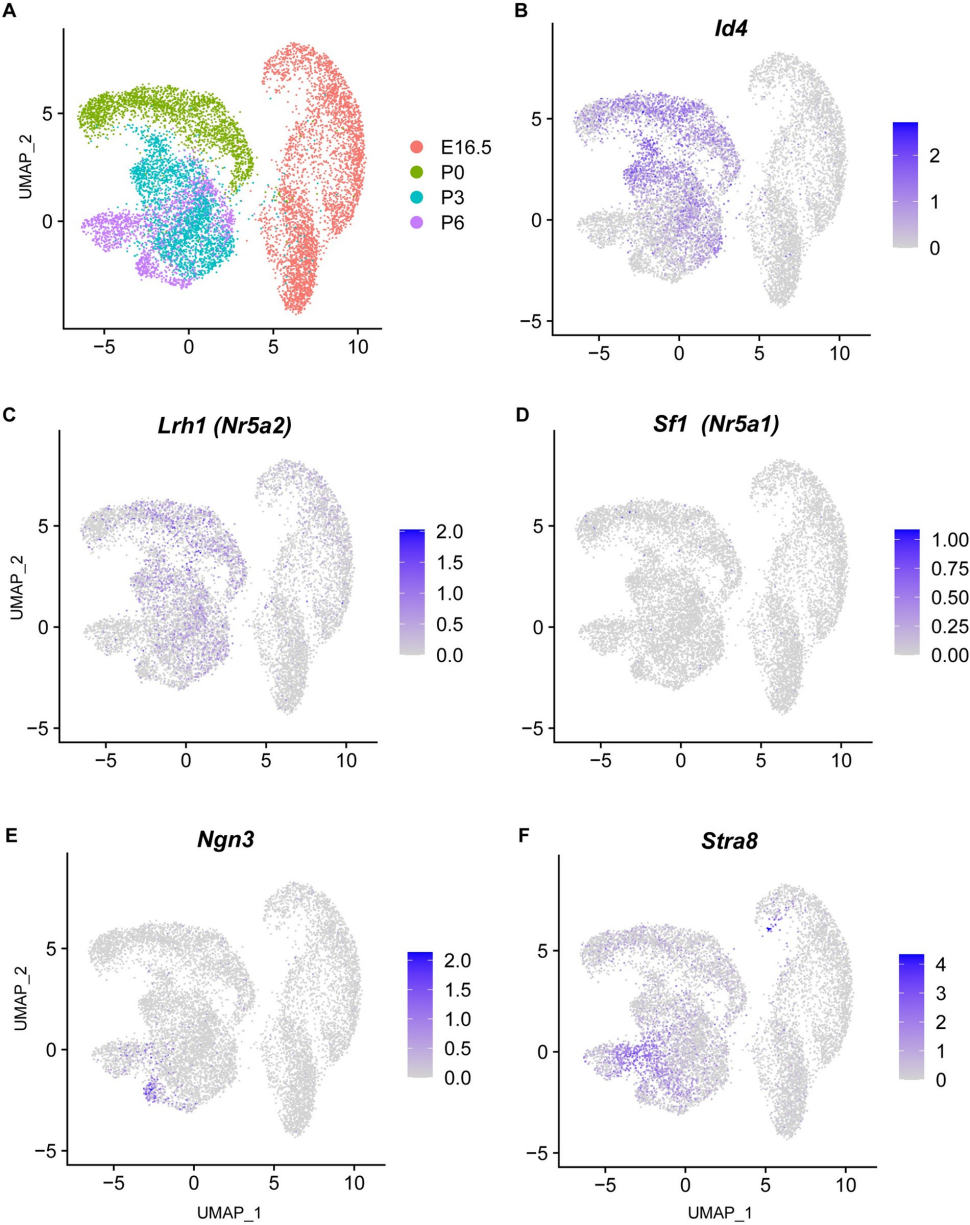
*Lrh1* is expressed in presumptive SSCs and undifferentiated spermatogonia. Analysis of scRNA-seq data of Law and colleagues [42]. (A) UMAP projection of data from prospermatogonia and spermatogonia isolated at E16 through P6 with developmental stages color coded as indicated in key. (B-F) Expression of *Id4*, *Lrh1*, *Sf1*, *Ngn3* and *Stra8* in isolated cells coded for expression level, with more intense blue color representing stronger expression.

To assess *Lrh1* expression in germ cells we analyzed single cell mRNA sequencing (scRNA-seq) data from fetal, juvenile, and adult testicular cells [42,43]. In germline scRNA-seq data from Law and colleagues [42], *Lrh1* was expressed at low levels in E16.5 prospermatogonia and higher levels in postnatal germ cells, mainly in presumptive SSC precursors also expressing *Id4* [44,45] (Fig 1A–1C). In contrast, *Sf1* was not significantly detected in germ cells at these stages (Fig 1D). We also compared *Lrh1* expression at these stages to that of the committed progenitor marker *Ngn3* [46], and the differentiation marker *Stra8* [47,48] (Fig 1E and 1F). At P3 and P6 *Lrh1* expression was higher in *Id4*-positive cells and low or absent in *Ngn3*-positive committed progenitor cells and in *Stra8*-positive differentiating spermatogonia. In summary, *Lrh1* expression in the juvenile germline was primarily in SSCs and undifferentiated spermatogonia rather than committed or differentiating spermatogonia. This view was confirmed by analysis of seminiferous epithelium scRNA-seq data of Hermann and colleagues [43], comparing expression of *Lrh1* and *Sf1* to that of a panel of somatic and germ cell markers (S1 and S2 Figs). At P6, as expected, *Sf1* was detected primarily in cells expressing the Sertoli cell marker *Sox9* and the Leydig cell marker *Cyp17a1*, while *Lrh1* expression was absent from Sertoli and Leydig cells (S1 Fig) [43]. At this stage *Lrh1* expression was detected mainly in germ cells expressing SSC markers including *Zbtb16*, *Etv5*, *Rhox10*, *Nanos2*, *Tspan8*, *Id4* and *Gfra1*. Expression was lower or absent in committed and differentiating spermatogonia marked by *Ngn3*, *Stra8*, *Dmrt1*, *Piwil2*, *Sohlh1*, *Sohlh2* and *Dmc1*, and in premeiotic cells strongly expressing *Stra8*. Expression of *Lrh1* was similar in the adult seminiferous epithelium, with expression primarily in undifferentiated spermatogonia including SSCs, and low or absent expression in differentiating spermatogonia and meiotic cells and in post-meiotic cells marked by *Prm1* (S2 Fig). Together these analyses indicated that *Lrh1* is expressed in juvenile Sertoli cells and in neonatal through adult SSCs and undifferentiated spermatogonia.

### LRH1 promotes Sertoli-to-granulosa reprogramming when *Dmrt1* is inactivated

**Fig 2:**
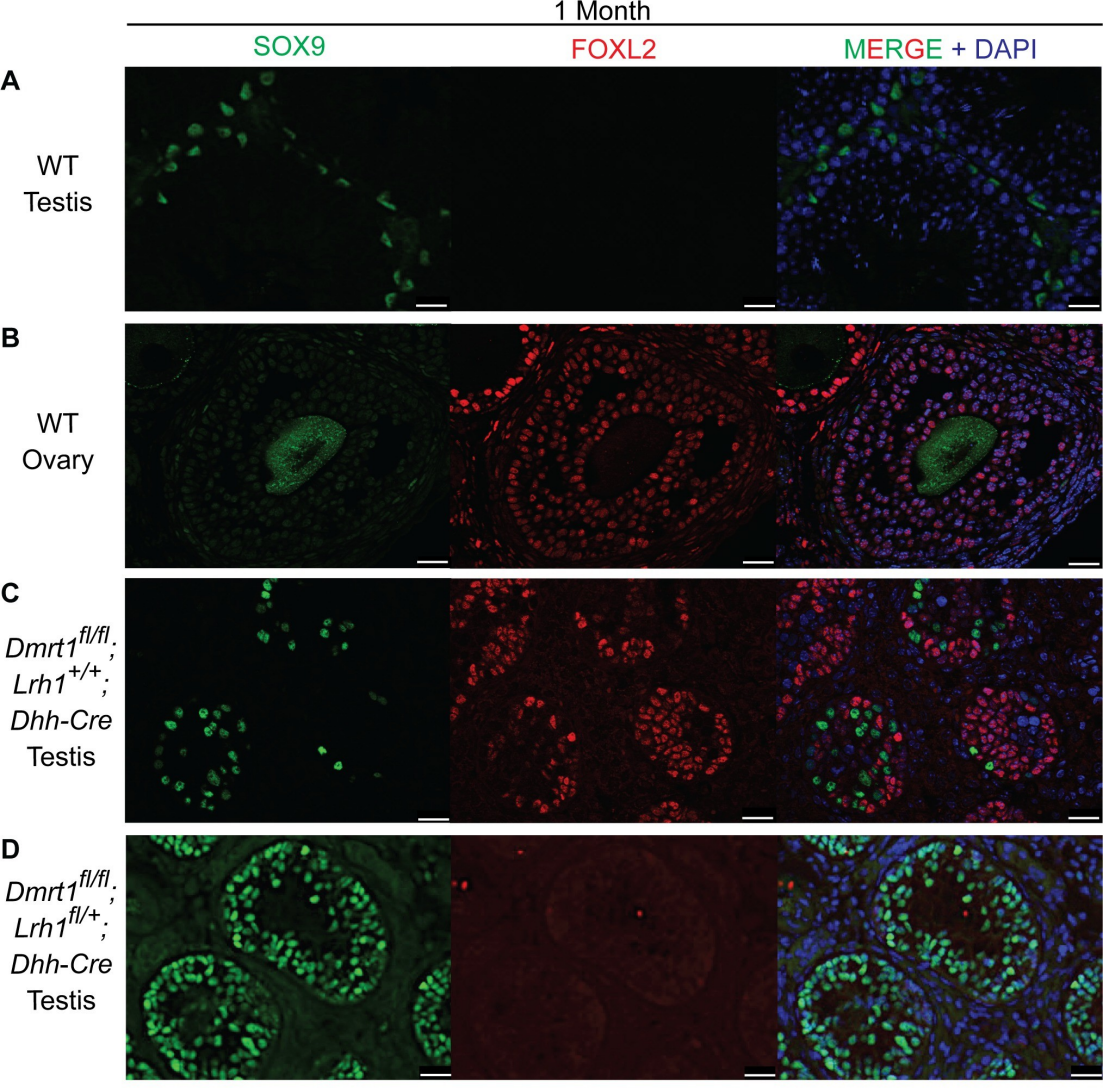
*Lrh1* is required for sexual cell fate transdifferentiation of *Dmrt1* mutant XY gonads. Immunofluorescence of gonads from one month old mice stained for the Sertoli cell marker SOX9 (green), the granulosa cell marker FOXL2 (red) and the DNA stain DAPI (blue). Genotypes shown are: (A) Wild type XY testis; (B) Wild type XX ovary; (C) XY gonad conditionally homozygous in Sertoli cells for *Dmrt1* deletion; (D) XY gonad conditionally homozygous in Sertoli cells for *Dmrt1* deletion and heterozygous for *Lrh1* deletion. Scale bars: 20 um.

Deletion of *Dmrt1* in Sertoli cells causes elevated expression of an array of female regulators including *Lrh1*, which is strongly upregulated in *Dmrt1* mutant XY gonads [3]. Expression of these regulators, many of which normally are expressed during fetal ovary determination and differentiation, reprograms Sertoli cells to granulosa-like cells starting late in the first postnatal week [3,49]. Genetic analysis has confirmed that some of these regulators, including *Foxl2*, *Esr2* and *Wnt* pathway components, are essential for Sertoli-to-granulosa transdifferentiation [49]. Although loss of *Lrh1* has not been shown to destabilize granulosa cell fate, *Lrh1* does play an essential role in granulosa cell differentiation [20]. We hypothesized, therefore, that strong overexpression of *Lrh1* might help promote the granulosa fate in *Dmrt1* mutant Sertoli cells. To test this possibility we deleted *Dmrt1* alone or together with *Lrh1* in fetal Sertoli cells using *Dhh-Cre* [50] to recombine “floxed” alleles of both genes. We then examined the gonads one month postnatally by immunofluorescence, asking whether deletion of *Lrh1* suppresses the transdifferentiation normally triggered by *Dmrt1* loss. SOX9 and FOXL2 are sex-specific regulators of somatic cell fate that can be used to assess sexual cell fate and transdifferentiation [3,4,49]. In wild type XY gonads, as expected, Sertoli cells expressed SOX9, and FOXL2 was not detected (Fig 2A), whereas in wild type XX gonads granulosa cells expressed FOXL2 and SOX9 was not detected, other than non-specific background staining of ooplasm (Fig 2B). As previously reported [3], deletion of *Dmrt1* in Sertoli cells results in loss of SOX9 expression and gain of FOXL2 expression in many cells as they adopt a female fate, together with depletion of male germ cells due to impaired somatic support. *Dmrt1* conditional mutant XY gonads had abundant strongly FOXL2-positive cells, much like wild type XX ovaries (Fig 2C), but deletion of *Dmrt1* together with even one copy of *Lrh1* in XY gonads suppressed ectopic FOXL2, resulting in virtually all targeted cells instead expressing SOX9 (Fig 2D). Because *Dmrt1* also is required not only for Sertoli fate maintenance but also for Sertoli cell maturation and support of spermatogenesis [51], the mutant gonads still lacked expanded tubule lumens and were severely depleted of germ cells (SOX9-negative cells with distinctive larger nuclei). Suppression of FOXL2 expression and rescue of SOX9 expression in *Dmrt1* mutant gonads depleted for *Lrh1* strongly suggests that LRH1 plays a crucial role in the reprogramming of Sertoli cell fate that occurs when DMRT1 is absent. Additionally, the nearly complete suppression of FOXL2 expression observed suggests that deletion of *Lrh1* in Sertoli cells by *Dhh-Cre* was highly efficient.

### Seminiferous tubule morphogenesis requires *Lrh1*

**Fig 3:**
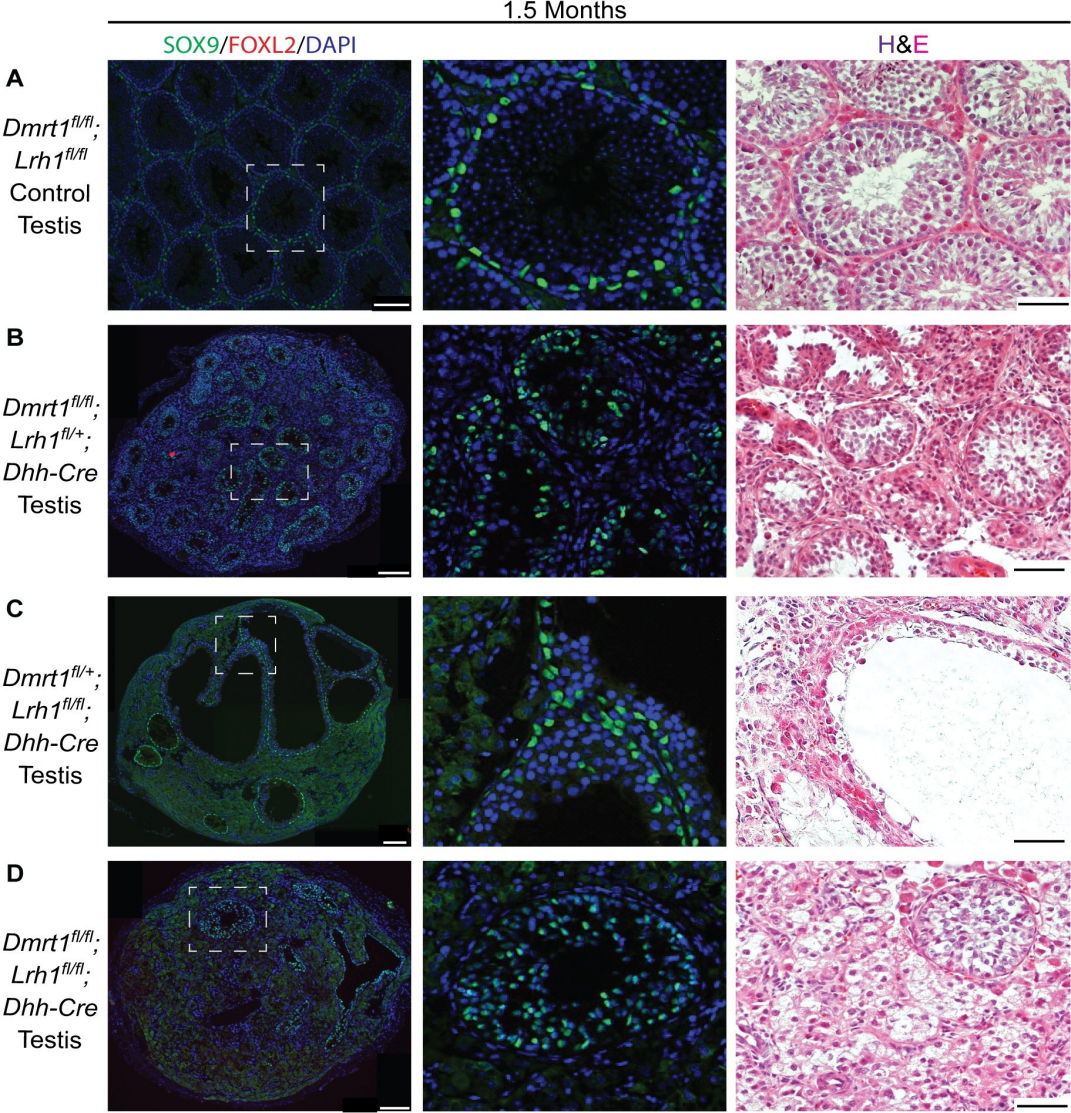
*Lrh1* is required in Sertoli cells for seminiferous tubule morphogenesis and spermatogenesis. Immunofluorescence (IF) and hematoxylin/eosin (H&E) staining for 6 week old testes of indicated genotypes: (A) Control testis homozygous for floxed alleles of *Dmrt1* and *Lrh1* but lacking *Cre*; (B) Mutant testis conditionally homozygous in Sertoli cells for *Dmrt1* deletion and heterozygous for *Lrh1* deletion; (C) Mutant testis conditionally heterozygous in Sertoli cells for *Dmrt1* deletion and conditionally homozygous for *Lrh1* deletion; (D) Mutant testis conditionally homozygous in Sertoli cells for both *Dmrt1* and *Lrh1* deletions. Dashed lines in left column indicate areas magnified in middle column. White scale bars: 100 um. Black scale bars: 50 um.

While reduced dosage of *Lrh1* in *Dmrt1* mutant XY gonads blocked transdifferentiation of Sertoli cells, gonads conditionally deleted for both genes also had greatly impaired seminiferous tubule morphogenesis (Fig 3). At 1.5 months, XY gonads conditionally mutant for *Dmrt1* and one allele of *Lrh1* expressed SOX9 and not FOXL2, as at one month, and they had smaller diameter tubules than controls lacking *Dhh-Cre*, at least partly due to depletion of germ cells (Fig 3A and 3B). Sertoli-specific loss of both *Lrh1* alleles together with one or both *Dmrt1* alleles (Fig 3C and 3D) more dramatically disrupted tubulogenesis, resulting in fewer and more highly disordered tubules. From these data we conclude that *Lrh1* expression not only plays a crucial role in the transdifferentiation of *Dmrt1* mutant Sertoli cells to granulosa-like cells but also is required for Sertoli cell support of germ cells and for normal seminiferous tubule morphogenesis.

### *Lrh1* is dispensable in Leydig cells for spermatogenesis and fertility

**Fig 4:**
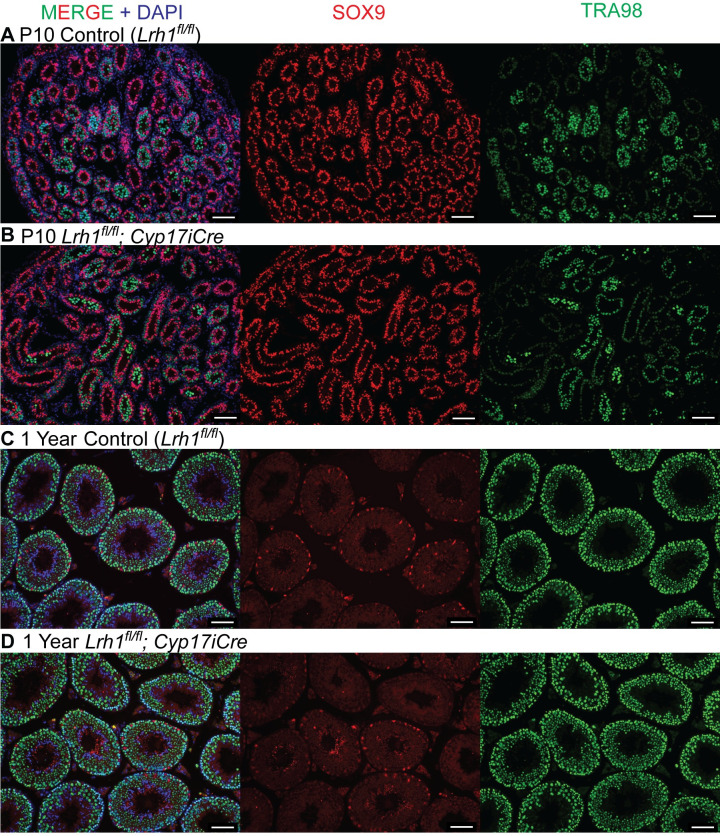
*Lrh1* is dispensable in Leydig cells. IF of control and Leydig cell conditional *Lrh1* homozygous mutant testes at P10 (A,B) and one year (C,D), stained for Sertoli cell marker SOX9 (red), germ cell marker TRA98 (green), and DNA marker DAPI (blue). Scale bars: 100 um.

**Fig 5:**
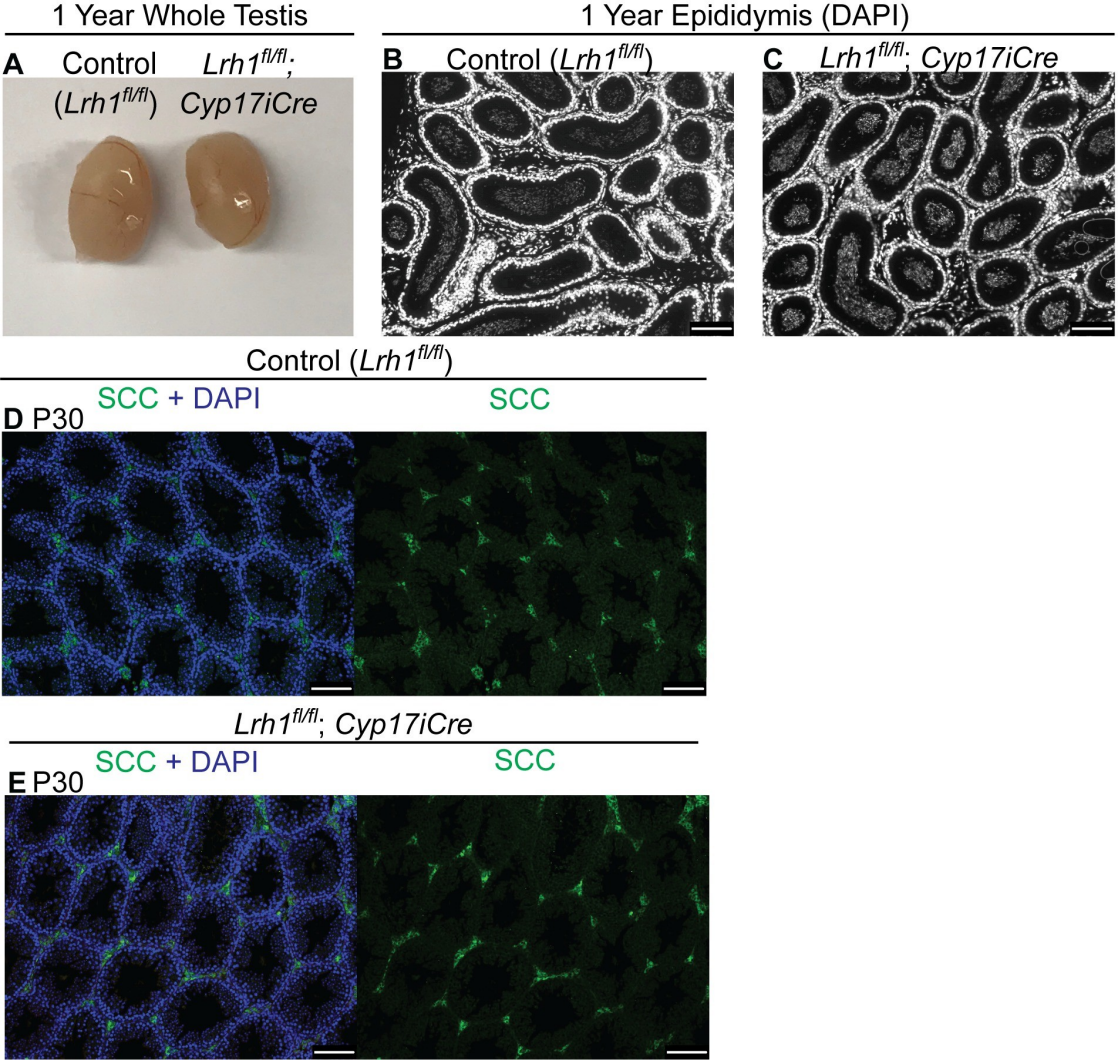
Normal testis size, spermatogenesis and Leydig cell development in Leydig cell conditional mutants. (A) Intact testes from one year control and Leydig cell conditional homozygous mutant. (B,C) DAPI fluorescence of epididymis from one year control and Leydig cell conditional mutant. (D,E) IF of control and Leydig cell conditional *Lrh1* mutant testes at P30 stained for Leydig cell marker SCC (green) and DAPI (blue). Scale bars: 100 um.

SF1 is essential for differentiation and function of Leydig cells [52]. Although mRNA profiling did not detect *Lrh1* expression in either fetal or adult Leydig cells, LRH1 can bind the same target DNA motif as SF1 [11–13], and we considered the possibility that *Lrh1* might be expressed at very low levels and play a role in Leydig cell development or function. We used *Cyp17iCre* [53] to delete *Lrh1* specifically in Leydig cells in the fetal testis, confirming deletion by postnatal testis PCR, and examined testis histology and morphology and male fertility between postnatal day 10 (P10) and one year (Figs 4 and 5). At both P10 (Fig 4A and 4B) and one year (Fig 4C and 4D), testis morphology appeared normal: germ cells (TRA98-positive), Sertoli cells (SOX9-positive) and Leydig cells (interstitial, TRA98/SOX9 negative cells) were present in normal numbers in conditionally targeted gonads relative to floxed controls lacking the *Cyp17iCre* transgene. Reproductive function also was normal in Leydig cell conditional mutants: testis size was unaffected at one year (Fig 5A); adult epididymi were filled with spermatozoa (Fig 5B and 5C); and conditionally-mutant males sired normal-sized litters. Conditionally-targeted Leydig cells also had normal expression of the key steroidogenic enzyme SCC (CYP11A1) (Fig 5D and 5E). Together with the lack of detectable *Lrh1* expression in Leydig cells, the lack of detectable phenotypes in Leydig-specific conditional mutants suggests that *Lrh1* is dispensable in Leydig cells for normal development and fertility.

### *Lrh1* is required in Sertoli cells for postnatal proliferation and gametogenesis

**Fig 6:**
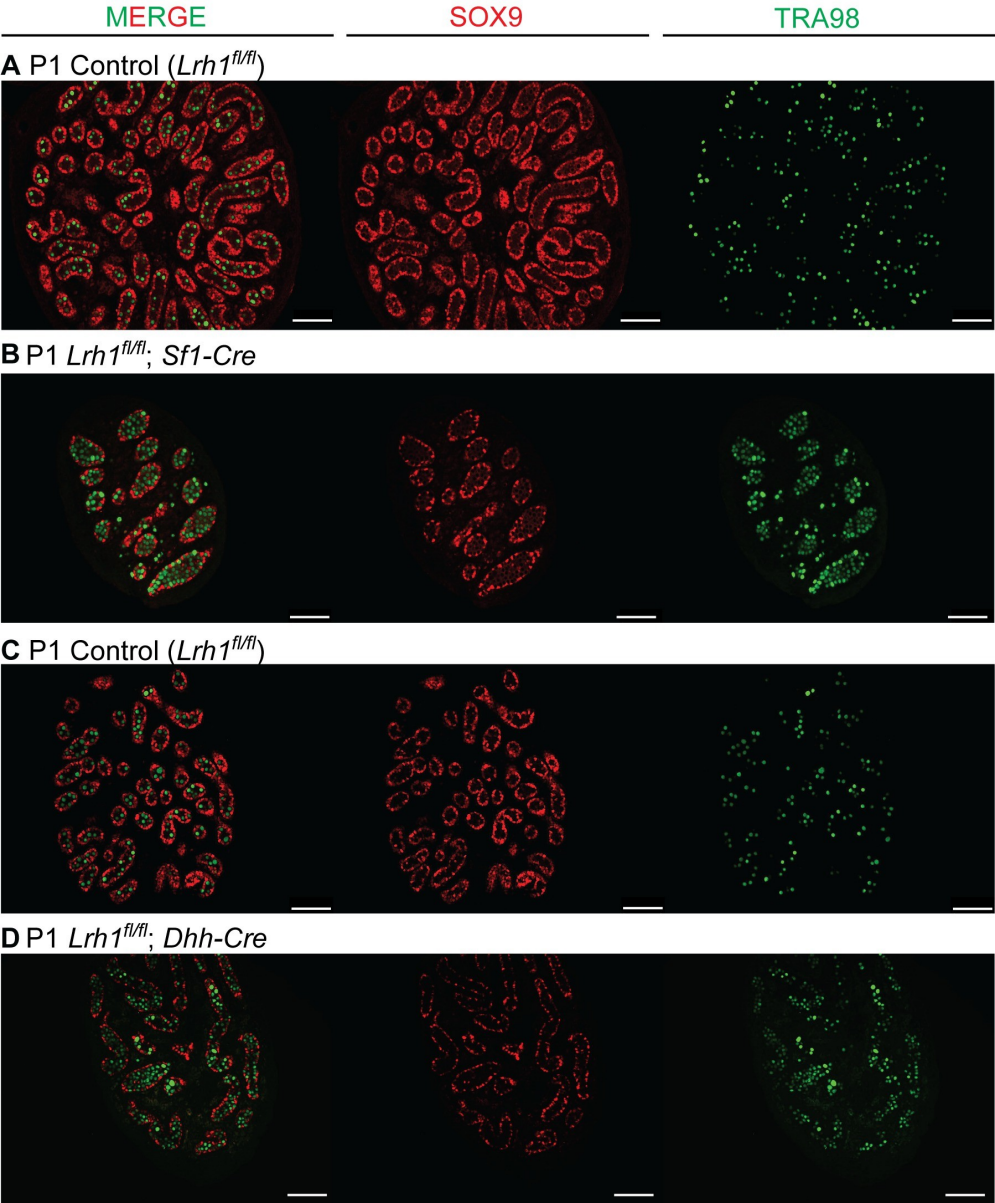
Fetal loss of *Lrh1* causes perinatal seminiferous tubule defects. (A,B) IF of P1 control and conditional *Lrh1* mutant testes deleted with *Sf1-Cre* and stained for SOX9 (red), TRA98 (Green) and DAPI (blue). (C,D) IF of P1 control and conditional *Lrh1* mutant testes deleted with *Dhh-Cre* and stained for SOX9 (red), TRA98 (Green) and DAPI (blue). Scale bars: 100 um.

**Fig 7:**
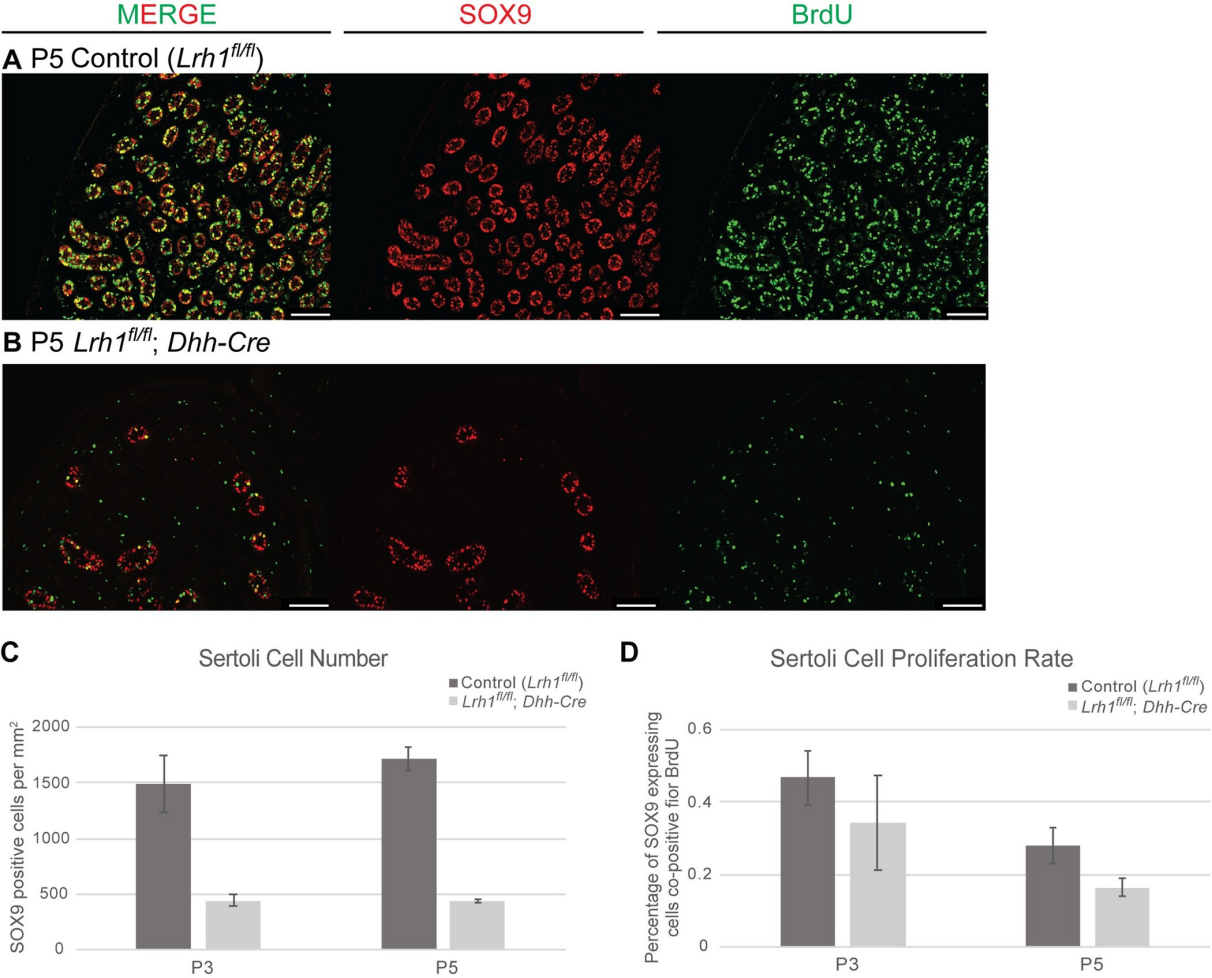
Fetal loss of *Lrh1* causes reduced perinatal Sertoli cell proliferation (A,B) IF of P5 control and conditional *Lrh1* mutant testes deleted with *Dhh-Cre* and stained for SOX9 (red), BrdU (green) and DAPI (blue). (C) Plot of SOX9-positive Sertoli cells per mm^2^ at P3 and P5 in control and *Dhh-Cre* deleted *Lrh1* conditional mutant testes. (D) Plot of BrdU-positive SOX9-expressing Sertoli cells in the same testes at P3 and P5. Error bars: standard error of mean (SEM). Scale bars: 100 um.

Because our conditional genetics indicated that function of *Lrh1* in the somatic gonad is primarily focused in Sertoli cells, we further examined this function. First we asked when loss of *Lrh1* in Sertoli cells first affects tubule morphogenesis using *Dhh-Cre*, which is active in differentiating fetal Sertoli cells [50], and *Sf1-Cre*, which is active in fetal Sertoli and Leydig cells [54]. Between E15.5 and E18.5 gonad size, tubule morphology and expression of the Sertoli markers SOX9 and TRA98 appeared unaffected by deletion of *Lrh1* with *Dhh-Cre* (S3A–S3D and S3G–S3J Fig). It was difficult to quantify the size of mutant tubules due to their irregular shapes but sectioning through the gonads showed qualitatively that mutant tubules were larger in diameter and less numerous by P0 (S3E, S3F, S3K and S3L Fig). Likewise, deletion of *Lrh1* using either *Dhh-Cre* or *Sf1-Cre* compromised tubulogenesis by P1, leading to smaller gonads with fewer tubules (Fig 6A–6D). More germ cells were located centrally in the mutant tubules, possibly due to a reduced number of Sertoli cells and thus less available tubule epithelium. These results suggest that tubulogenesis defects arise perinatally. At P5 *Dhh-Cre* conditional mutant testes remained small and appeared to have reduced numbers of germ cells (Fig 7A and 7B). (*Sf1-Cre* conditional mutants died after P1, presumably due to consequences of extragonadal deletion.) To assess Sertoli cell numbers and proliferation in *Dhh-Cre* conditional mutants we quantified the number of SOX9-positive Sertoli cells per area and the proportion of BrdU-positive Sertoli cells at P3 and P5 (Fig 7C and 7D). We performed two-way ANOVA fit followed by post-hoc Tukey’s honest significance difference test. The number of Sertoli cells per area was affected by loss of *Lrh1* (p = 5.18 x 10^−5^) but not by age (p = 0.441). Proliferation rate determined by the fraction of BrdU-positive cells was affected by both loss of *Lrh1* (p = 0.0291) and by age (p = 0.00988). We also examined Sertoli cell death by TUNEL labeling and did not observe any increase in P0 or P3 mutant testes. Together these results indicate that *Lrh1* promotes expansion of Sertoli cell numbers during the previously described perinatal proliferative period [55].

**Fig 8:**
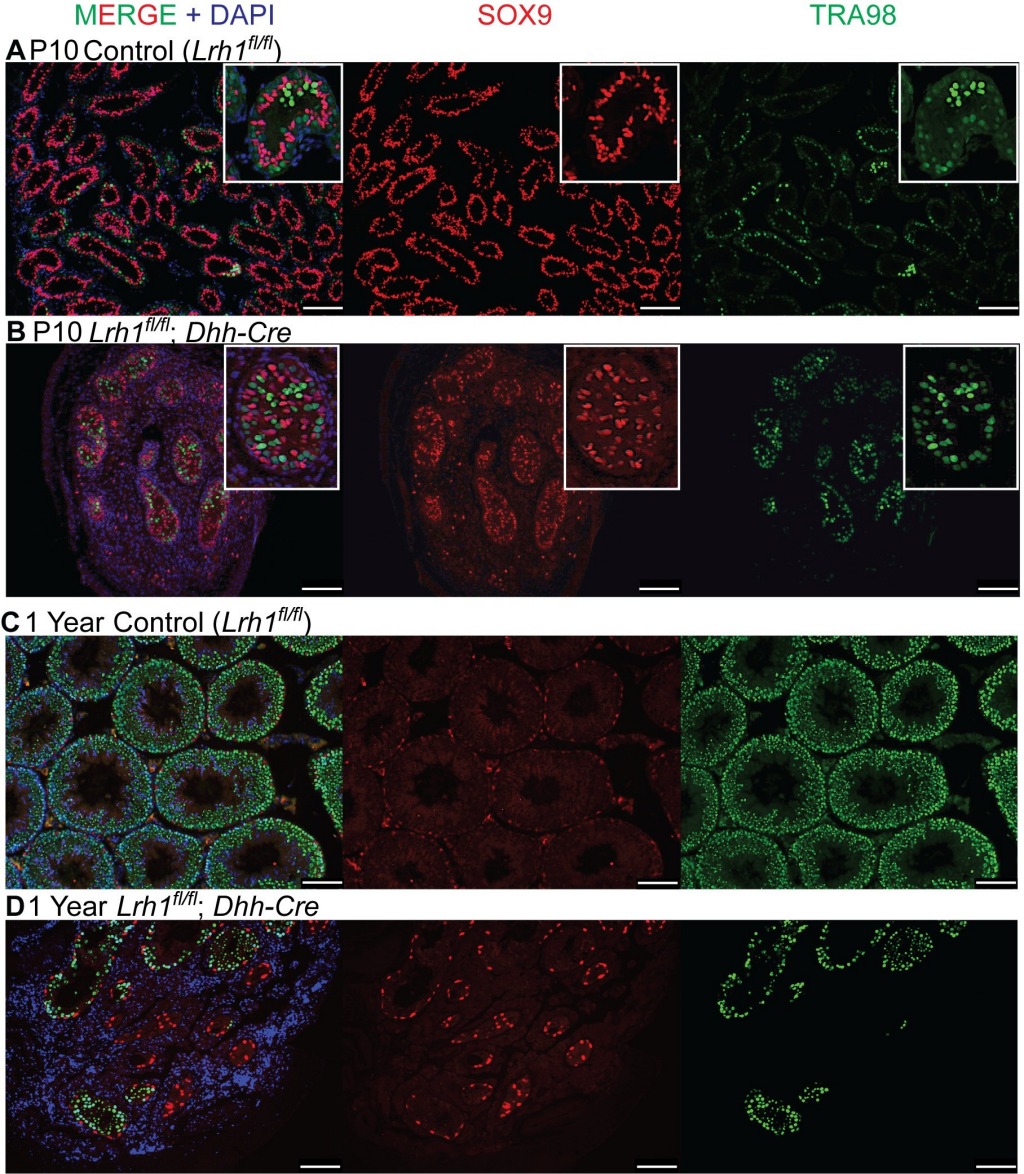
*Lrh1* is required in Sertoli cells for sustained and robust spermatogenesis. IF of control and *Dhh-Cre* deleted Sertoli-specific *Lrh1* conditional mutant testis sections stained for SOX9 (red), TRA98 (green) and DNA (DAPI, blue). (A,B) P10 testes, showing reduced testis size and disorganized tubules; (C,D) one year testes, showing presence of presence and absence of spermatogenesis in disorganized tubules. Scale bars: 100 um.

**Fig 9:**
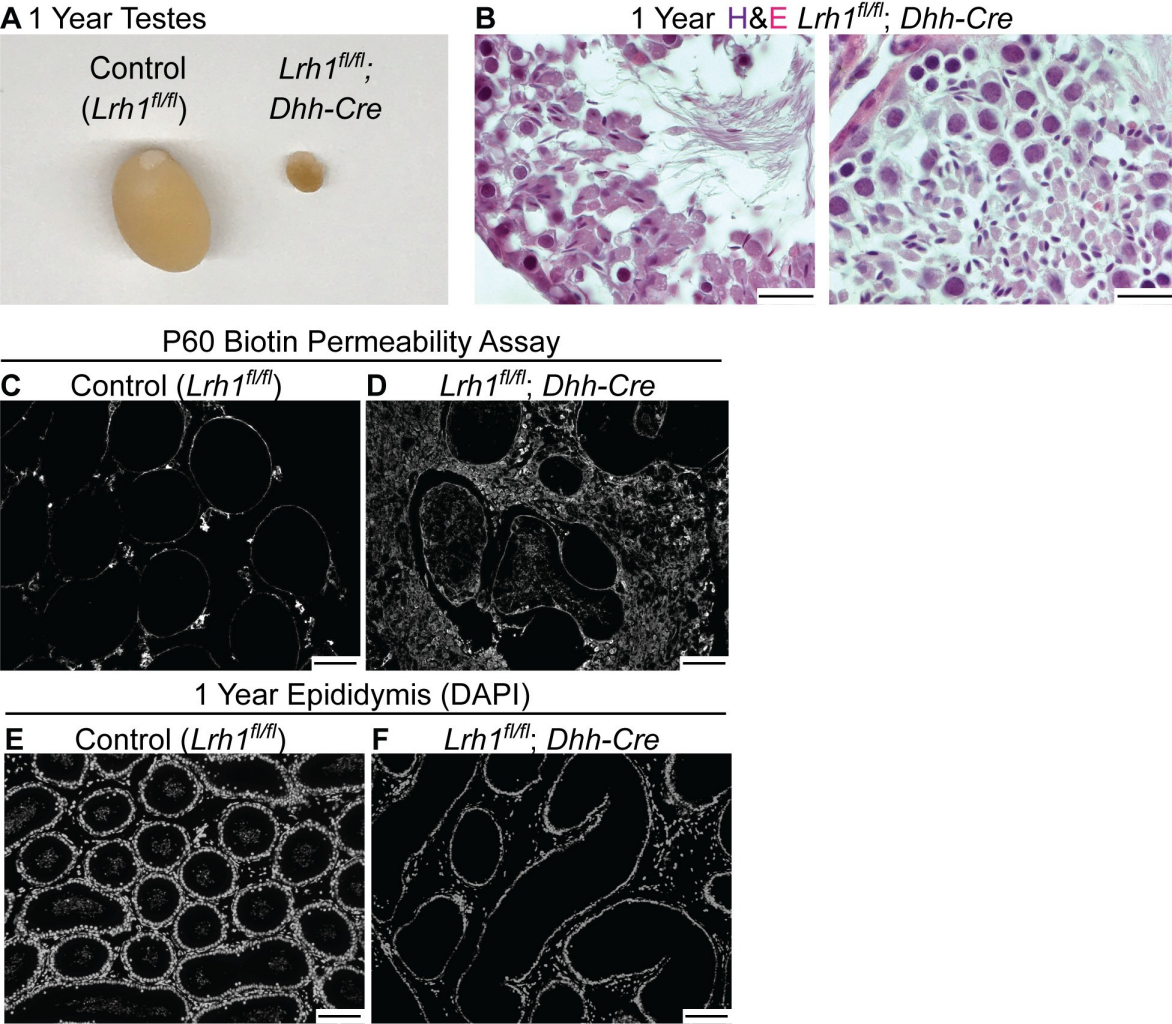
*Lrh1* is required in Sertoli cells for normal testis size and blood-testis barrier integrity. (A) Comparison of intact control and *Dhh-Cre*-deleted testes at one year showing severe hypoplasia in mutant. (B) H&E stained sections of one year old *Dhh-Cre* conditional *Lrh1* mutant testis sections showing presence of elongated and mature spermatids in a minority of tubule sections. (C,D) Streptavidin stained testis sections from biotin-injected control and *Dhh-Cre* conditional *Lrh1* mutant showing abnormal permeability of mutant tubules. (E,F) DAPI stained epididymis sections from control and *Dhh-Cre* conditional *Lrh1* mutant showing absence of epididymal sperm in mutant. Scale bars: 20 um in panel B; 100 um in panels C-F.

We also asked whether the disrupted tubule morphology in Sertoli cell conditional mutants severely disrupts gametogenesis. At P10, testes with *Lrh1* conditionally deleted in Sertoli cells were small and had dysgenic tubules in which Sertoli and germ cells were present but many were not properly localized to the tubule periphery (Fig 8A and 8B). At one year most tubules were small, irregular, and severely depleted of TRA98-positive germ cells, although some tubule sections did retain germ cells (Fig 8C and 8D). Tubules were malformed and testis size was severely reduced relative to controls (Fig 9A). H&E staining confirmed that a small number of sections contained elongated spermatids and mature spermatozoa (Fig 9B), suggesting that *Lrh1* mutant Sertoli cells were able to support complete gametogenesis, albeit with greatly reduced efficiency.

Sertoli cell tight junctions form the blood-testis barrier (BTB), which is present from puberty onward and is essential for spermatogenesis because it protects meiotic and postmeiotic germ cells from immune surveillance [56]. We investigated the possibility that the disorganized tubules might have a compromised BTB that contributes to failure of gametogenesis. To test BTB function we assayed permeability of tubules to interstitially-injected biotin and found all tubules in the control excluded biotin but many tubules in the mutant gonads were biotin-permeable (Fig 9C and 9D). We did observe some biotin-impermeable tubules that lacked germ cells, suggesting that a compromised BTB is likely not the sole cause of the gametogenesis defects in mutants. Despite the presence of spermatids in the mutant testis we did not detect any spermatozoa in the epididymis of conditional mutants (Fig 9E and 9F). This absence might result from the very low numbers of mature testicular spermatozoa or from inefficient transport of spermatozoa from the disorganized mutant testis into the epididymis. As expected from the lack of epididymal sperm, conditional mutant males were unable to sire progeny when mated to wild type females.

**Fig 10:**
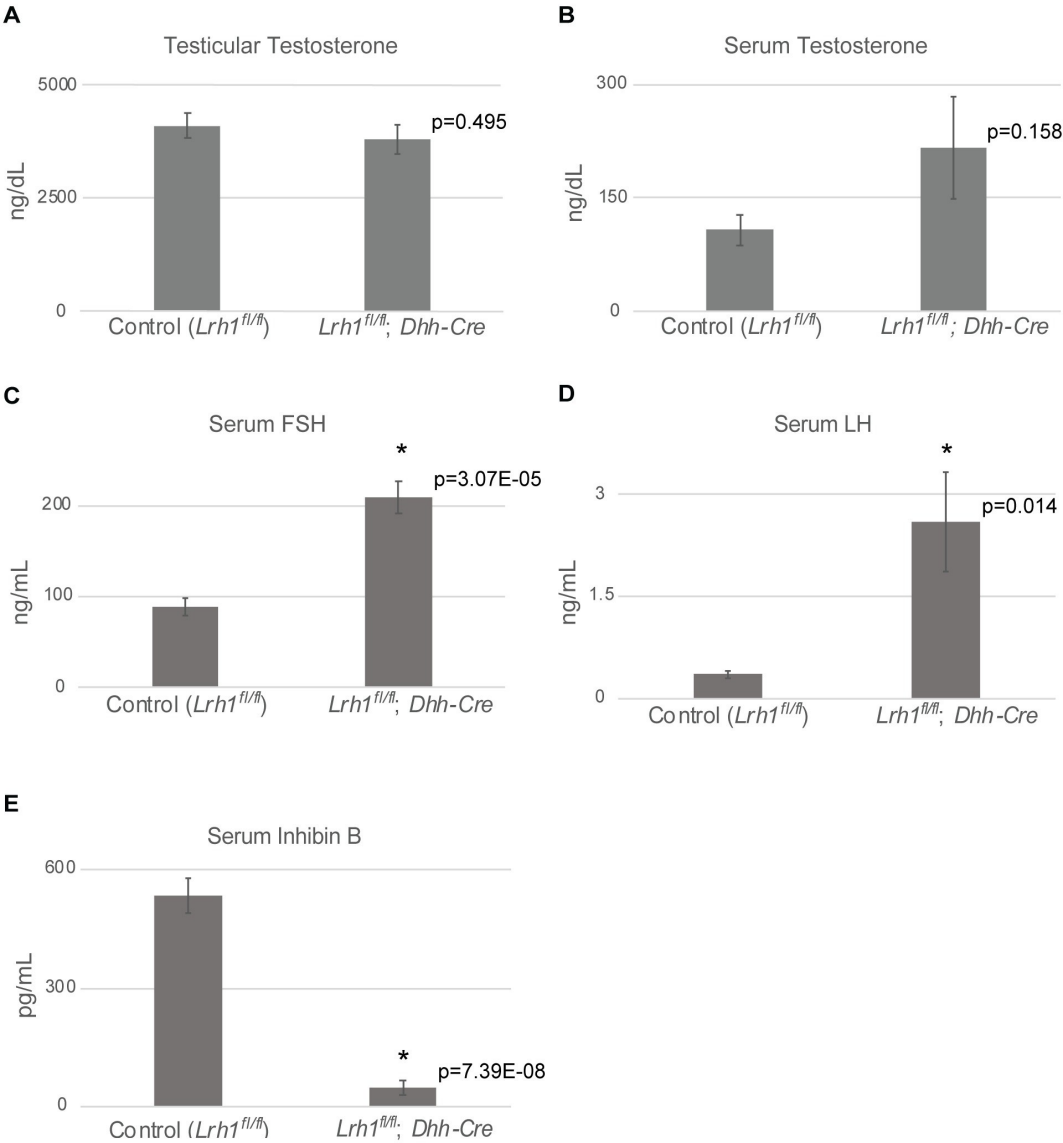
*Lrh1* is required in Sertoli cells for feedback regulation of the hypothalamic-pituitary axis. Plots show mean and standard deviation for hormone levels in control (N = 12) and *Dhh-Cre* conditional *Lrh1* mutant (N = 10) testes (A) or serum (B-E). Asterisks indicate statistically significant difference. Error bars: SEM.

Sertoli cells play a central role in gonadal hormone production, so we evaluated testicular and circulating hormone levels in Sertoli-specific *Lrh1* mutant males (Fig 10). We found that total testosterone levels were normal in testes of conditional mutants (3803 ng/dl, versus 4097 ng/dl in controls; p-value = 0.495). Average circulating testosterone was higher in mutants by about two-fold, 216 versus 107 ng/dl, but the difference was not statistically significant (p-value = 0.158; Fig 10A and 10B). Serum FSH was elevated about two-fold (from 88 to 208 ng/ml) with a highly significant p-value (p = 3.07 x 10^−5^), and LH was elevated about eight-fold (from 0.35 to 2.6 ng/ml), also with a significant p-value (p = 0.014; Fig 10C and 10D). Sertoli cells normally limit FSH and LH release from the pituitary by feedback regulation; the elevated levels of these hormones suggested a possible defect in this feedback. We therefore assayed circulating inhibin B, a key feedback signal from Sertoli cells, and found that it was reduced about ten-fold, from 533 to 46 pg/ml (Fig 10E; p-value = 7.39 x 10^−8^). Together these results suggest that at least some of the defects caused by *Lrh1* loss in Sertoli cells may derive from failure of inhibin-mediated feedback to the hypothalamic-pituitary-gonadal axis.

To learn more about the nature of Sertoli cell defects in mutants we performed RNA-seq on testes from Sertoli-specific mutants at P3. Because mutant testes have 3-fold fewer Sertoli cells at this stage we only examined genes with >3-fold misexpression (S1 Table). To ensure that expression changes were robust we excluded genes with expression of less than 0.5 FPKM in control testes. This analysis identified 77 downregulated and 48 upregulated genes. Among these genes were a number with functions that potentially contribute to the *Lrh1* mutant phenotype (Table 1). For example, downregulated genes have been shown to control Sertoli cell metabolism (*Foxq1*), cell adhesion and tight junctions (*Cdh23*, *Cgn*) and tubule organization (*Erbb4*). Sex hormone binding globulin (*Shbg*), which functions to retain androgens in the testis [57], also was downregulated. Among the upregulated were genes involved in blood-testis barrier regulation (*Tgfb2*), signaling regulation of SSC self-renewal (*Nrg1*), Sertoli cell energy metabolism and proliferation (*Ngf*), and Sertoli cell proliferation (*Cdkn1a*). The enlarged tubules in conditional mutant testes were reminiscent of those reported in testes mutant for the estrogen receptor gene *Esr1* [58], suggesting a possible defect in estrogen signaling via ESR1. However, while *Esr2* expression was reduced at P3, *Esr1* expression was not significantly altered.

**Table 1:** Genes linked to reproductive function that are regulated by *Lrh1* in Sertoli cells.

Gene	Fold change	Reported functions	References (PMID)
*Etd*	-5.78	Novel testis-specific gene	12617826
*Foxq1*	-5.70	Forkhead TF; required for lactate metabolism in Sertoli cells and for spermatogenesis	34091745
*GM2044*	-5.46	lncRNA expressed in spermatocytes	29934815
*Shbg*	-5.02	Sex hormone binding globulin; expressed in Sertoli cells, required for testosterone retention	21613632
*Cited1*	-4.54	P300-interacting transactivator expressed in Sertoli cells	28459107
*Masp1*	-4.47	Sertoli-enriched serine peptidase; regulated by Inhibin A.	32274496
*Cstdc1*	-4.41	Sertoli-expressed cystatin-related protein; also called *CstSC*	18391548
*Cdh23*	-4.29	Cadherin-23; mediates cell adhesion	30747484
*Spp1*	-4.23	Secreted phosphoprotein; expressed in Sertoli cells and spermatogonia; also called *Osteopontin*	15937924, 18240954
*Gata1*	-3.73	Sertoli-specific GATA TF; regulates *Inhbb*	12954777, 11075815
*Pak6*	-3.71	Testis-expressed p21-activated kinase; interacts with androgen and estrogen receptors	11278661, 11773441
*Rasd1*	-3.61	Ras-related protein; may mediate Sertoli proliferation	29162477
*Sfrp4*	-3.53	Secreted frizzled-related protein; regulated by Inhibin A	32274496
*Aard*	-3.51	Alanine- and arginine-rich domain protein; regulated by androgen receptor in Sertoli cells	17486547, 17959439
*Sox9*	-3.34	Sry-related TF; required for establishment of Sertoli cell fate	15056615
*Cgn*	-3.32	Tight junction protein	26821949
*Esr2*	-3.23	Estrogen receptor; may regulate Sertoli cell differentiation	24056172
*Erbb4*	-3.05	Receptor tyrosine kinase; required in Sertoli cells for tight junctions and tubule organization	25058600
*Lrrc4c*	4.53	Leucine rich repeat protein; copy number variation associated with human infertility	25439847
*Nrg1*	3.86	Neuregulin secreted signaling protein; regulates Sertoli proliferation, promotes SSC self-renewal	30040984, 25504872
*Tgfb2*	3.48	TGF-beta signaling ligand; disrupts blood-testis barrier	18192323
*Ngf*	3.34	Nerve growth factor; regulates Sertoli cell energy metabolism and proliferation	28470720, 32567558
*Cdkn1a*	3.32	Cell cycle regulator, also called *p21Cip1*; regulates Sertoli cell proliferation; LRH1 represses *Cdkn1a* to stimulate proliferation in multiple cell types	15728790, 26400164, 25435372
*Cyp1b1*	3.18	P450 enzyme; stimulated by FSH and LH; regulated by LRH1	19074971, 25873311

### *Lrh1* is required for SSC maintenance but not for recovery from germ cell depletion

**Fig 11:**
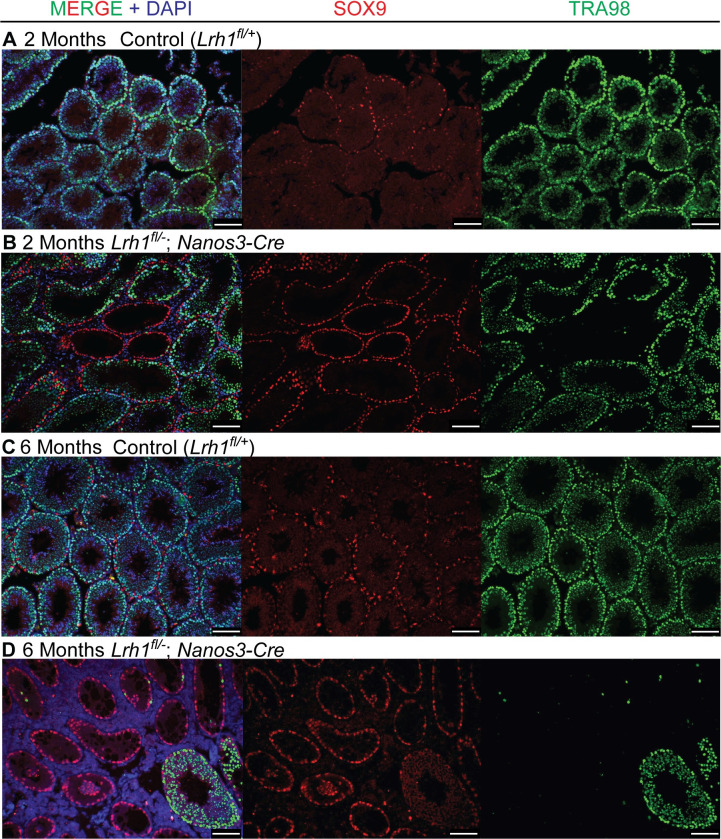
Loss of *Lrh1* in germ cells causes progressive loss of spermatogenesis. IF of sections from control or germ cell-specific *Nanos3-Cre Lrh1* mutant testes stained for SOX9 (red), TRA98 (green) and DAPI (blue) at 2 months (A,B) and 6 months (C,D). Scale bars: 100 um.

**Fig 12:**
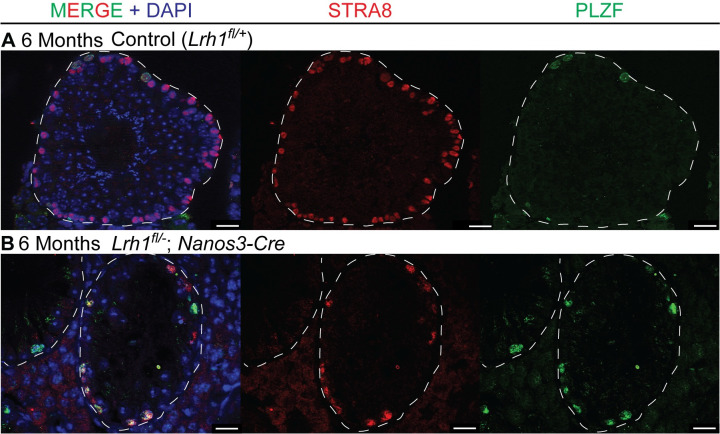
Loss of *Lrh1* causes failure of spermatogonial differentiation. IF of sections from control (A) or germ cell-specific *Nanos3-Cre Lrh1* mutant (B) testes stained for STRA8 (red), PLZF (green) and DAPI (blue) at 6 months, showing persistence of spermatogonia with depletion of differentiated germ cells. Scale bars: 100 um.

Conditional deletion in somatic cells showed that *Lrh1* is required cell non-autonomously in Sertoli cells for spermatogenesis. Next we asked whether *Lrh1* also is autonomously required in the germ line. We used *Nanos3-Cre* [59] to delete *Lrh1* in fetal germ cells from about E12.5 and examined germ line development from birth. We found that conditional mutant testes appeared normal for about one month postnatally but then underwent a progressive loss of spermatogenesis, with most tubule sections severely or completely depleted of germ cells by six months (Fig 11A–11D). Most tubule sections lacked germ cells entirely, suggesting a defect in SSC maintenance. In other depleted tubules there were small numbers of germ cells remaining. These cells had typical spermatogonial morphology were a mixture of cells expressing the undifferentiated spermatogonial marker PLZF and cells also expressing the differentiating spermatogonial marker STRA8 (Fig 12A and 12B). This pattern and the lack of more advanced germ cell types in most tubule sections suggests that mutants are unable to maintain a functional population of SSCs and spermatogonia. Testes were very small by six months and no sperm were detected in the epididymis at this stage (S4A and S4B Fig).

**Fig 13:**
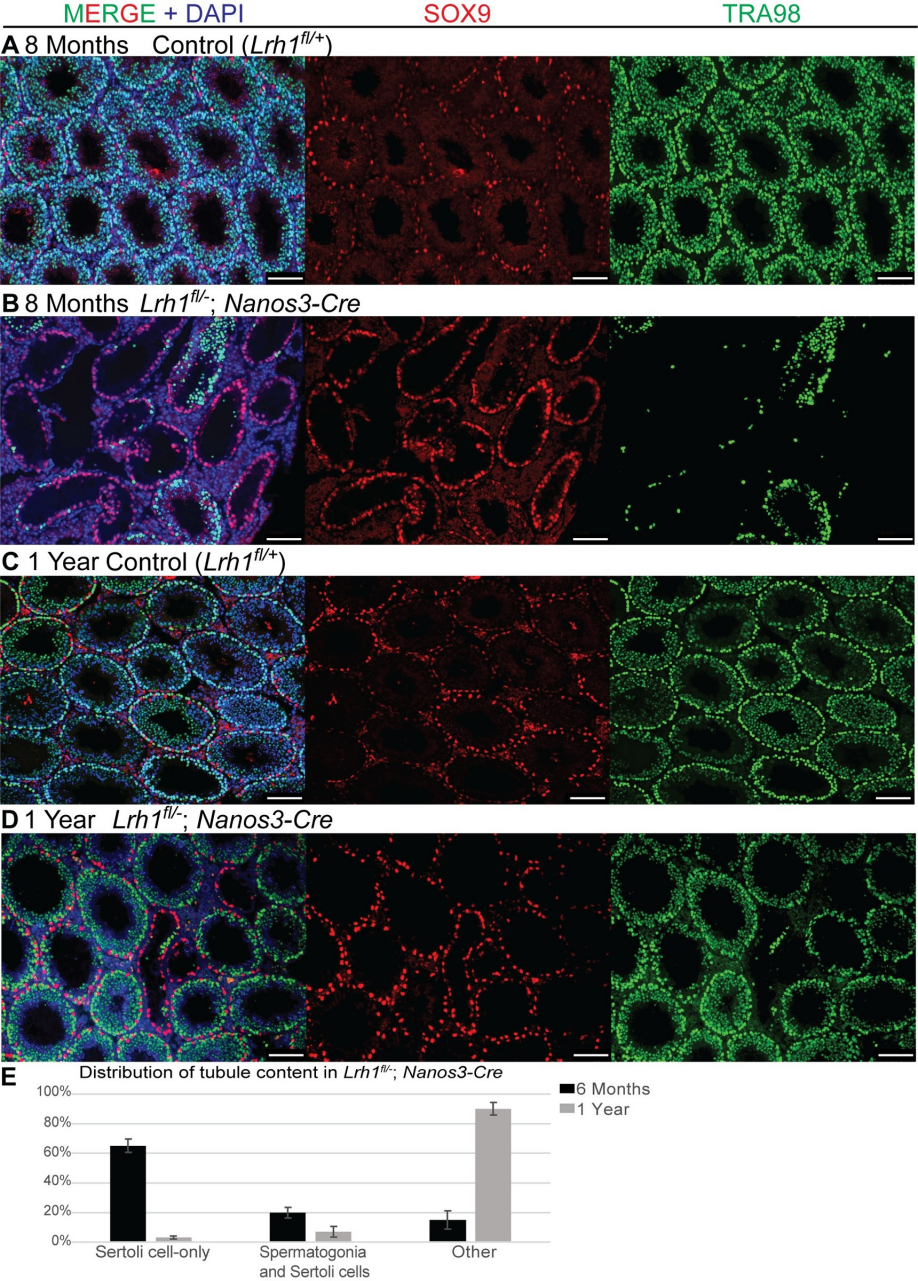
Recovery of spermatogenesis in *Lrh1* germ cell mutant testes. (A-D) IF of sections from control or germ cell-specific *Nanos3-Cre Lrh1* mutant testes stained for SOX9 (red), TRA98 (green) and DAPI (blue) at eight months (A,B) and one year (C,D). Scale bars: 100 um. (E) Plot of germ cell composition of seminiferous tubules in germ cell mutant testes at six months and one year. Error bars: SEM.

Unexpectedly, we found that spermatogenesis recovered in conditional mutant testes after six months, with recovery evident by eight months and nearly all tubules showing substantial spermatogenic cell profiles by one year (Figs 13A–13E and S5E) and spermatozoa reappearing in the epididymis by 8 months (S5G–S5I Fig). Progressive loss of spermatogenesis also occurred in heterozygous conditional mutants, but was delayed by several months relative to homozygotes; abundant germ cell deficient tubules appeared around eight months instead of six months, and severe depletion was evident at one year, a stage by which homozygous mutants had recovered abundant spermatogenesis (S5A–S5F Fig). At 8 months, heterozygous conditional mutants had greatly reduced epididymal sperm relative to controls and homozygotes (S5G and S5H Fig) and at one year, heterozygous conditional mutants had severely reduced testis size compared with controls and homozygous mutants (S6A–S6C Fig), and still had very few epididymal sperm, in contrast to the abundant sperm in controls and homozygous mutants (S6D–S6F Fig). The progressive loss of spermatogenesis was again consistent with a defect in spermatogonial function.

We considered possible explanations for the recovery of spermatogenesis in germ cell-specific *Lrh1* mutants. A trivial explanation is that deletion of *Lrh1* was not highly efficient and wild type cells remained that were able to repopulate the depleted tubules. To test this possibility, we bred three homozygous conditional mutant males to wild type females starting at one year of age, after spermatogenesis had recovered. We genotyped the offspring, asking whether the deleted *Lrh1* allele was efficiently transmitted by mutant males. All offspring from eleven litters (89/89) were heterozygous for *Lrh1*, indicating that more than 98% of functional germ cells in the conditionally mutant males carried the mutant allele. We therefore conclude that germ line deletion of *Lrh1* was highly efficient but surviving mutant germ cells can somehow be activated to re-establish spermatogenesis after severe depletion.

To learn more about the role of *Lrh1* in spermatogonia we performed RNA-seq comparing germ line conditional mutant testes with heterozygous controls lacking *Nanos3-Cre* at P1, seeking the earliest changes in gene expression that might underlie the later phenotype. We detected significant misexpression of only four genes at this stage in conditional mutants relative to controls lacking *Cre*: expression of the mitochondrial CoA transporter *Slc25a42*, the C-type lectin *Chodl*, and the cerebellin1 precursor *Cbln1* was elevated, while expression of the nuclear RNA export factor *Nxf2* was reduced (S1 Table). Of these four genes, only *Nxf2* has been reported to function in the testis. NXF2 is restricted to spermatogonia and spermatocytes within the testis, with nuclear localization in spermatogonia and localization to the nuclear periphery in spermatocytes [60]. Strikingly, deletion of *Nxf2* in mice has revealed two functions: the gene is required for spermatogonial proliferation and SSC maintenance, and also, in a strain-dependent manner, for meiotic progression [61]. Since *Nxf2* mutant mice exhibit age-dependent loss of spermatogenesis similar to that observed in *Lrh1* germ cell-specific mutants [61], reduced *Nxf2* expression might play a role in the *Lrh1* germ line phenotype.

## Discussion

Here we have used conditional genetics to explore the function of *Lrh1* in the mouse testis. We find that *Lrh1*, like its close paralog *Sf1*, plays an essential role in testis development but, unlike *Sf1*, *Lrh1* is required both in the somatic and germ cell lineages. Analysis of published RNA sequencing data showed that *Lrh1* mRNA is expressed in neonatal and juvenile Sertoli cells and in juvenile and adult spermatogonia, but not in Leydig cells. Conditional gene targeting revealed that *Lrh1* is essential in Sertoli cells for their normal development and function and is required in the germ line for SSC maintenance or function. Consistent with its lack of expression in this cell type, we found that *Lrh1* does not appear to have an essential function in Leydig cells. In Sertoli cells *Lrh1* was required for several distinct functions including neonatal proliferation, seminiferous tubule morphogenesis, support of spermatogenesis, blood-testis barrier integrity, and hormonal feedback to the hypothalamus/pituitary axis. In the germ line *Lrh1* was required for sustained spermatogenesis, and the progressive loss of spermatogenesis in mutants, including loss of spermatogonia, suggests a defect in SSC maintenance, differentiation, or both. Profiling of mRNA in mutant testes identified a number of potential mediators of *Lrh1* action in Sertoli cells and germ cells, some with well-established functions and some that are candidates for further analysis.

We also examined the role of *Lrh1* in sexual cell fate reprogramming. These experiments were motivated by the previous observation that *Lrh1* is highly overexpressed in XY testes mutant for *Dmrt1* and undergoing male-to-female transdifferentiation [3]. Loss of *Lrh1* strongly suppressed the formation of FOXL2-positive granulosa-like cells in *Dmrt1* mutant XY testes, indicating that *Lrh1* expression is required for this cell fate transformation. The mechanism by which *Lrh1* promotes cell fate reprogramming in this context is unknown. It is noteworthy, however, that LRH1 can replace OCT4 in reprogramming cultured cells to pluripotency [36]. One possibility, therefore, is that LRH1 helps drive Sertoli cells to a more permissive and plastic chromatin state that can be more readily reprogrammed by other factors that are active when DMRT1 is missing.

We found similarities and differences in the roles of *Lrh1* and its close paralog *Sf1* in the testis. As summarized earlier, *Sf1* plays important roles in development of both Sertoli and Leydig cells but appears to affect germ cell development only indirectly. In contrast, we find that *Lrh1* is important and acts cell type autonomously in Sertoli cells and germ cells but is dispensable in Leydig cells. *Sf1* is important for the earliest stages of fetal Sertoli cell determination and development. In contrast, *Lrh1* appears to be required in Sertoli cells only from late fetal or perinatal stages. Both genes regulate sex hormone levels but they do so differently. *Sf1* controls steroidogenesis in Leydig cells by controlling expression of key steroidogenic enzymes. Our results suggest that *Lrh1* acts on steroidogenesis mainly via Sertoli cell feedback control of gonadotropins.

Loss of *Lrh1* in the germ line caused a classic progressive failure of spermatogenesis that is suggestive of compromised SSC maintenance or differentiation. Strikingly, however, spermatogenesis recovered in the mutant gonads, and older mutant males were fertile. In heterozygous mutants spermatogenesis also failed progressively, but with a delay of several months relative to homozygotes, and it did not recover by one year. We confirmed, by breeding older homozygous males, that the gametes after recovery were mutant for *Lrh1* and were not simply cells that escaped targeting. Since the repopulating germ cells were mutant, a puzzle is what allows spermatogenesis to recover after depletion, given that *Lrh1* clearly is required in germ cells for sustained spermatogenesis prior to depletion. One idea is that germ line depletion reduces a signaling molecule needed for spermatogenesis but, beyond a critical threshold of depletion, Sertoli cells can respond by producing this molecule to restart spermatogenesis. A strong candidate for the signaling molecule would be retinoic acid (RA), which normally is produced by Sertoli cells in the juvenile testis to initiate spermatogonial differentiation and meiosis and later is produced by spermatocytes to help sustain steady state spermatogenesis [62]. Reactivated RA production in Sertoli cells might be sufficient to stimulate developmentally arrested *Lrh1* mutant spermatogonia to differentiate when spermatocytes and other advanced germ cells are severely depleted. Another scenario, which is also speculative, is that a surviving population of quiescent “reserve” SSCs can be activated when germ cell depletion reaches a critical threshold and that these cells do not require *Lrh1* for proliferation or differentiation. In either scenario, heterozygotes would fail to recover by one year because they reach the critical depletion threshold later than homozygotes. Testing these and other possible mechanisms will be a future challenge but our analysis documents a remarkable ability of the germ line to respond to and functionally recover from a severe genetic disruption.

This study establishes *Lrh1* as a key regulator of testis development and function, performing essential functions both in somatic and germ line cells of the testis. We recently identified sex-biased differentially accessible regions (DARs) in Sertoli and granulosa cells that are likely to mediate sex-biased gene expression, and we found that these regions are enriched for the DNA motif bound by SF1 and LRH1 [63]. Because LRH1 had no reported function in the testis, we suggested that these sites might be bound by SF1. Given the important role of LRH1 in Sertoli cells revealed by the present study, these sites also are candidates for LRH1 binding. It will be informative to perform comparative ChIP-seq or CUT&RUN analysis to learn which sites are bound by each protein in each cell type and whether they control separate or overlapping sets of target genes. These and other comparative approaches will help establish the relationship between this pair of nuclear hormone receptors in supporting male reproduction.

## Materials and methods

### Ethics statement

Experimental protocols and euthanasia protocols were approved by the University of Minnesota Animal Care and Use Committee (protocol number: 2106-39153A).

### Mice

Mice were of mixed genetic background (129Sv and C57Bl/6J) and maintained in conventional housing facilities. Presence of a copulation plug in the morning was recorded as day E0.5.

*Dhh-Cre* mice [50] were provided by D. Meier, *Sf1-Cre* mice [54] were provided by K. Parker, *Nanos3-Cre* mice [59] were provided by Y. Saga, *Cyp17iCre* mice [53] were provided by C. Mendelsohn and M. Griswold. *Lrh1* floxed mice [37] were provided by K. Schoonjans and J. Auwerx. For Sertoli and Leydig cell conditional gene targeting, males homozygous for the *Lrh1* floxed allele were bred to females homozygous for the *Lrh1* floxed allele and carrying a cell type-specific *Cre* transgene. Homozygous floxed offspring lacking *Cre* were used as controls for *Dhh-Cre* and *Sf1-Cre* experiments. Heterozygous floxed/wild type animals lacking *Cre* were used as controls for *Nanos3-Cre* phenotypic analysis and floxed/null animals lacking Cre were used as controls for *Nanos3-Cre* RNA-seq experiments. In all experiments, gonads from a minimum of three animals were examined for each genotype at each developmental stage described.

### Genotyping

PCR genotyping on tail clip DNA for the *Lrh1* floxed allele transgene was conducted using primers: 5’ (5’ GCT ATA GGG AGT CAG GAT ACC ATG G 3’) and 3’ (5’ GTT CGT ACC ACT TTC ATC TCC TCA CG 3’) which yield an approximately 300 bp band for the wild type allele and a 400 bp band for the floxed allele. Reactions ran for 35 cycles with a 30 second 60°C annealing step and a 1-minute 72°C elongation. To genotype the *Lrh1* deleted allele the *Lrh1* 5’ primer was used with the deletion 3’ primer (5’ TCT GCA CAGCAG AAA ACC TCC 3’). 10% betaine was included in PCR reactions. Reactions ran 35 cycles with a 1 minute 60°C annealing step and 1 minute 72°C elongation. The deletion allele band is approximately 350 bp. Genotyping of the *Dmrt1* floxed allele, and the *Cyp17iCre*, *Nanos3-Cre*, *Dhh-Cre*, and *Sf1-Cre* transgenes were as described [50,51,53,54,64].

### Immunofluorescent staining

Immunofluorescence was performed on paraffin-embedded 5μm tissue sections after fixation with Bouin’s fixative (Sigma HT10132) or 4% paraformaldehyde (Electron Microscope Sciences 15710) diluted in PBS. Slides were stained overnight after antigen retrieval. Slides were blocked using donkey serum (S30-100ML Sigma-Aldrich) diluted to 10% in 1X PBS with 0.1% BSA (Sigma A9647) and 0.1% Tween 20 (Sigma P9416). The following primary antibodies were used: rabbit anti-DMRT1 (1:400) [65]; rat anti-TRA98 (1:200) (Abcam ab82527); rabbit anti-SCC (1:200) (Chemicon AB1294); goat anti-GFP (1:600) (Novus NB100-1770); rat anti-BrdU (Abcam ab6326), rabbit anti-SOX9 (1:200) (EMD Millipore AB5535); rabbit anti-RFP (1:200) (Rockland 600401379); and goat anti-FOXL2 (1:200) (Abcam ab5096). The following secondary antibodies were used: Alexa Fluor 594 donkey anti-rabbit IgG (Invitrogen A21207); Alexa Fluor 488 donkey anti-rabbit IgG (Invitrogen A21206); Alexa Fluor 594 donkey anti-rat IgG (Invitrogen A21209); Alexa Fluor 488 donkey anti-rat IgG (Invitrogen A21208); Alexa Fluor 594 donkey anti-goat IgG (Life Technologies A11058); and Alexa Fluor 488 donkey anti-goat IgG (Invitrogen A11055). Slides were counterstained with DAPI diluted in PBS at 1 mg/ml (Thermo Scientific 62248) to detect nuclei. Slides were cover slipped (Fisher Scientific 22037298) in mounting medium (Permafluor, Thermo Scientific TA030FM).

### Image capture

Immunofluoresence images were captured with a Zeiss Axio Imager Z1 microscope and Zeiss MRm camera, processed and false-colored using Zeiss Axiovision software. High magnification images were captured using Zeiss Apotome structured illumination. Histology (hematoxylin and eosin) images were captured with a Leica DMRB microscope using a Zeiss MRc camera and processed using Zeiss MRGrab software.

### Sertoli cell numbers and BrdU incorporation

BrdU was injected intraperitoneally two hours prior to tissue collection and detected using rat anti-BrdU antibody (Abcam ab6326), as described [66]. SOX9-positive Sertoli cells with and without BrdU incorporation were counted from testis cross-sections separated by at least 20 um, with three cross-sections counted per animal and three animals per age and genotype. Testis section area was measured and used to normalize Sertoli cell numbers per square millimeter.

### BTB permeability assay

Biotin permeability was assayed essentially as described [67,68] using freshly dissected testes from P60 mutant and control. Ten mg/ml of EZ-Link Sulfo-NHS-LC-Biotin (Pierce 21335) diluted in 1×PBS containing 1 mM CaCl_2_ was injected into one testis while the other was injected with vehicle. Testis sections were stained with Alexa Fluor 594 conjugated streptavidin (Invitrogen S32356).

### Mating test

Three one-year-old *Lrh1*^*fl/-*^; *Nanos3-Cre* males were bred to three fertile C57B6/J wildtype females. Eighty-nine offspring from eleven litters (three or more litters per male) were genotyped to ensure that the *Lrh1* allele was deleted in male germ cells. All male and female offspring were found to be heterozygous for *Lrh1*, confirming transmission of the mutant allele.

### Hormone measurements

Testicular and blood serum testosterone measurements, blood serum luteinizing hormone (LH), blood serum follicle stimulating hormone (FSH), and blood serum inhibin-B for control and mutant mice were performed by The University of Virginia Center for Research in Reproduction Ligand Assay and Analysis Core. The intra-assay coefficient of variation for testicular testosterone measurements ranged from 0.4 to 10.5% and for serum measurements ranged from 0.1 to 19.2% for testosterone, 0 to 10% for LH, 0.3 to 8.9% for FSH, and 1.5 to 19.4% for inhibin-B. Significance between the control and mutant assays was determined using a Two-sample Assuming Unequal Variances t-test. Samples analyzed were from twelve control and ten mutant mice. Six controls were eliminated from LH assays and four from inhibin-B assays due to sub-threshold values.

### Analysis of seminiferous tubule content

Round tubule cross sections were examined from at least six testis sections separated by at least 20 um from at least three animals per age and genotype (73–334 tubule sections per section). Tubules were classified as being Sertoli-only, containing spermatogonia, or containing both spermatogonia and more advanced germ cells (“other”). Statistical comparison was by paired Student’s t-test.

### mRNA sequencing

P1 or P3 testes were harvested and homogenized in Trizol reagent (Thermo Fisher) and stored at -80°C prior to processing. Total RNA was extracted from the aqueous phase, mixed with ethanol and purified using the RNeasy kit protocols and reagents (Qiagen). RNA was quantified using the Qubit RNA assay (Thermo Fisher) and 400–500 ng total RNA per sample was used in stranded mRNA-seq library preparation (KAPA Biosystems, KK8481) for Illumina sequencing. Libraries were pooled and sequenced with 2x150 cycles paired-end to an average depth of 26 million reads per sample on a HiSeq 4000 by Genewiz (South Plainfield, NJ). Reads were trimmed using Trim Galore (v0.6.0) and cutadapt (v1.18) and assessed for quality with FastQC (v0.11.8). Trimmed reads were mapped with STAR (v2.7.2a) to the GRCm38 (mm10) genome. The GENCODE M25 gene annotation set was used to estimate strand-specific gene expression data with the Bioconductor package RSubread (v2.2.6). Differentially expressed genes were identified using DESeq2 (v1.28.1).

## Supporting information

S1 Fig *Lrh1* expression in P6 seminiferous epithelium. UMAP projection of scRNA-seq data from Hermann and colleagues [43]. Level of expression in single cells is represented by blue color intensity.
(TIF)

S2 Fig *Lrh1* expression in adult seminiferous epithelium. UMAP projection of scRNA-seq data from Hermann and colleagues [43]. Level of expression in single cells is represented by blue color intensity.
(TIF)

S3 Fig Sertoli-specific *Lrh1* conditional mutant testes develop tubulogenesis defects perinatally. (A-F) IF staining of testis sections for Sertoli marker SOX9 (green) and DAPI (blue) comparing controls lacking *Cre* and *Dhh-Cre* deleted *Lrh1* mutants. (A,B) E15.5, (C,D) E18.5, (E,F). P0 (G-L) IF staining of testis sections for germ cell marker TRA98 (green) and DAPI (blue) comparing controls lacking Cre and *Dhh-Cre* deleted *Lrh1* mutants. (G,H) E15.5, (I,J) E18.5, (K,L) P0. Scale bars: 50 um.
(TIF)

S4 Fig *Lrh1* germ cell conditional mutants have small testes and severely depleted epididymal sperm at 6 months. (A) Intact testes from control and *Nanos3-Cre* deleted germ cell conditional mutant at 6 months. (B,C) H&E stained sections of epididymi from control and germ cell conditional mutants. Scale bars: 50 um.
(TIF)

S5 Fig *Lrh1* heterozygous germ cell conditional mutants have delayed loss of spermatogenesis relative to homozygotes. (A-D) IF of testis sections stained for SOX9 (red), TRA98 (green) and DAPI (blue) showing presence of both Sertoli-only and spermatogenic tubules at 8 months (A,B) and mainly Sertoli-only tubules at 1 year (C,D). (E,F) Plots comparing percentage of seminiferous tubules with normal spermatogenesis in *Lrh1* heterozygous and homozygous germ cell conditional mutant testes (E) and distribution of tubule germ cell content (F) from 6 months to one year. (“Other” indicates mix of spermatogonia and advanced germ cells.) Error bars: SEM. (G-I) H&E stained sections of epididymi from control and germ cell conditional *Lrh1* mutants showing abundant epididymal sperm in homozygous mutant (I) but not in heterozygous mutant (H) at 8 months. White scale bars: 100 um. Black scale bars: 50 um.
(TIF)

S6 Fig Spermatogenesis recovers in homozygous but not heterozygous germ cell conditional *Lrh1* mutants by one year. (A) *Lrh1* homozygous *Nanos3-Cre* deleted germ cell mutant testes are normal size and heterozygotes are severely reduced in size relative to controls at one year. (B-C) Plots showing observed testis size of collected heterozygous and homozygous mutant mice at 6 months, 8 months, and 1 year. (D-F) H&E stained sections of epididymi from control and germ cell conditional mutants showing abundant epididymal sperm in homozygous mutant (F) but not in heterozygous mutant (E) at 1 year. Scale bars: 50 um.
(TIF)

S1 Table mRNA profiling data. Sheet 1: expression level (FPKM) of genes expressed in each experiment. Sheet 2: differentially expressed genes in Nanos-Cre deleted *Lrh1* mutant testes at P1. Sheet 3: differentially expressed genes in *Dhh-Cre* deleted *Lrh1* mutant testes at P3.
(XLSX)
