# Supplementary figures and images for: Lrh1 can help reprogram sexual cell fate and is required for Sertoli cell development and spermatogenesis in the mouse testis
Source: PLoS Genet. 2022 Feb 22;18(2):e1010088. doi: 10.1371/journal.pgen.1010088 (PMC8896720; doi:10.1371/journal.pgen.1010088)

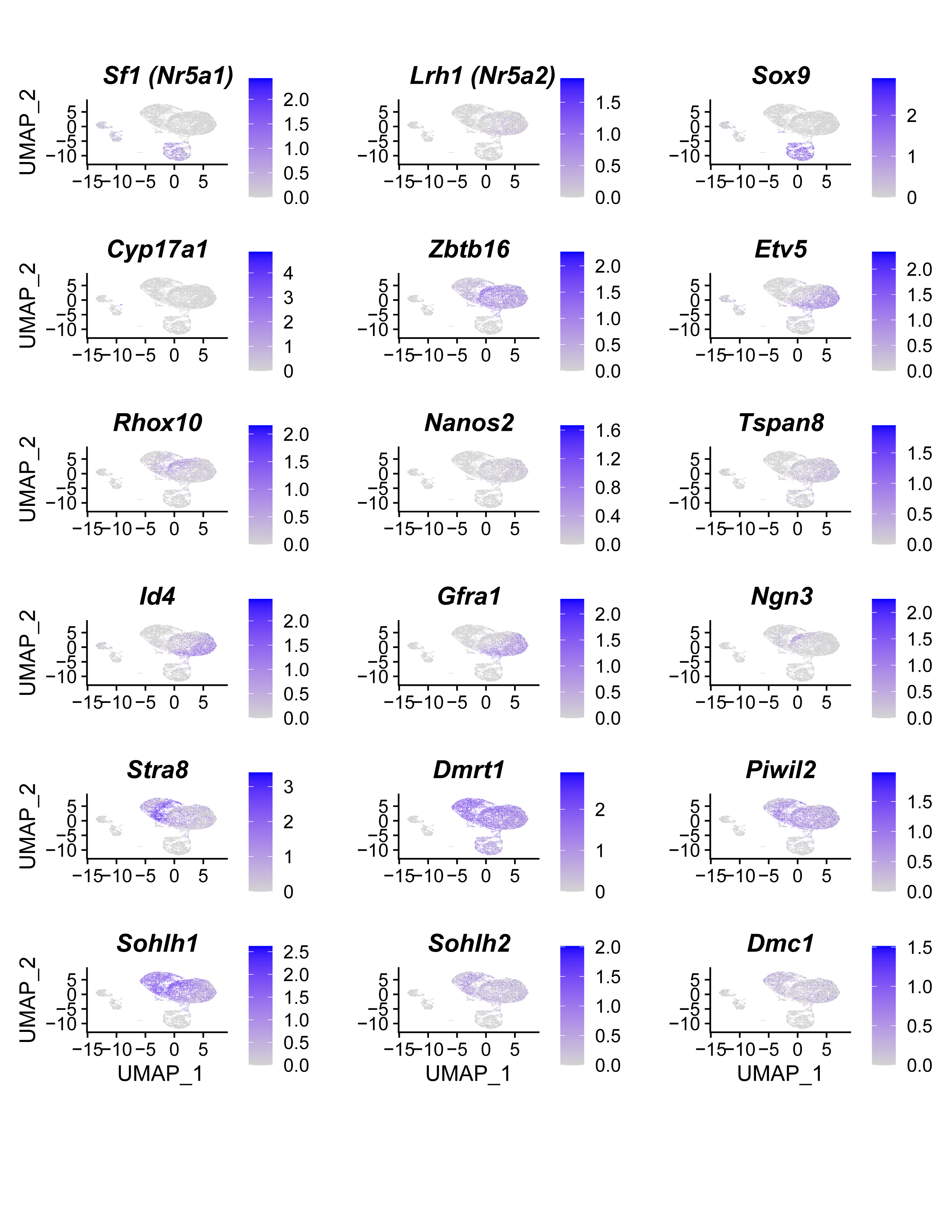

Supplement: S1 Fig — UMAP projection of scRNA-seq data from Hermann and colleagues [43]. Level of expression in single cells is represented by blue color intensity. (TIF) [file pgen.1010088.s001.tif]

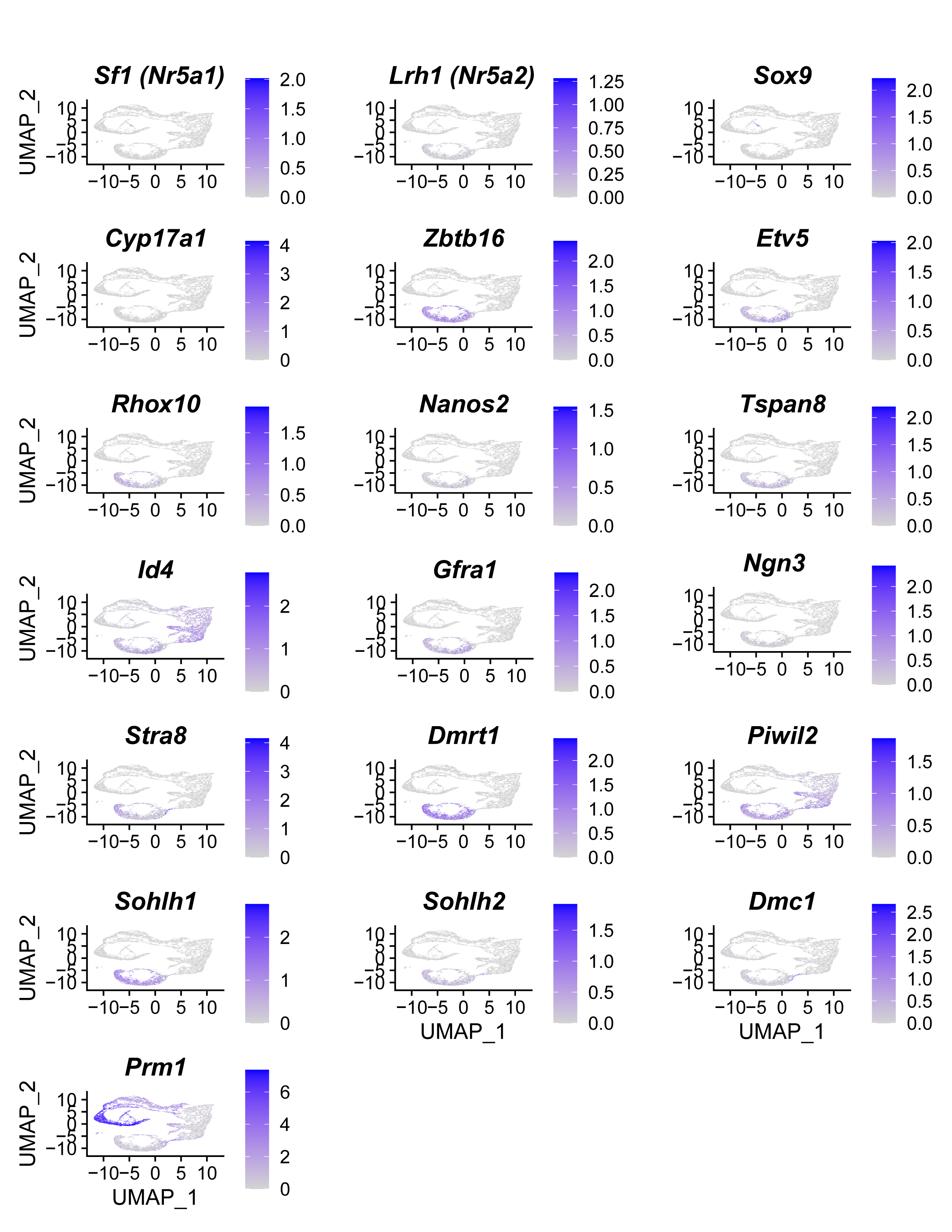

Supplement: S2 Fig — UMAP projection of scRNA-seq data from Hermann and colleagues [43]. Level of expression in single cells is represented by blue color intensity. (TIF) [file pgen.1010088.s002.tif]

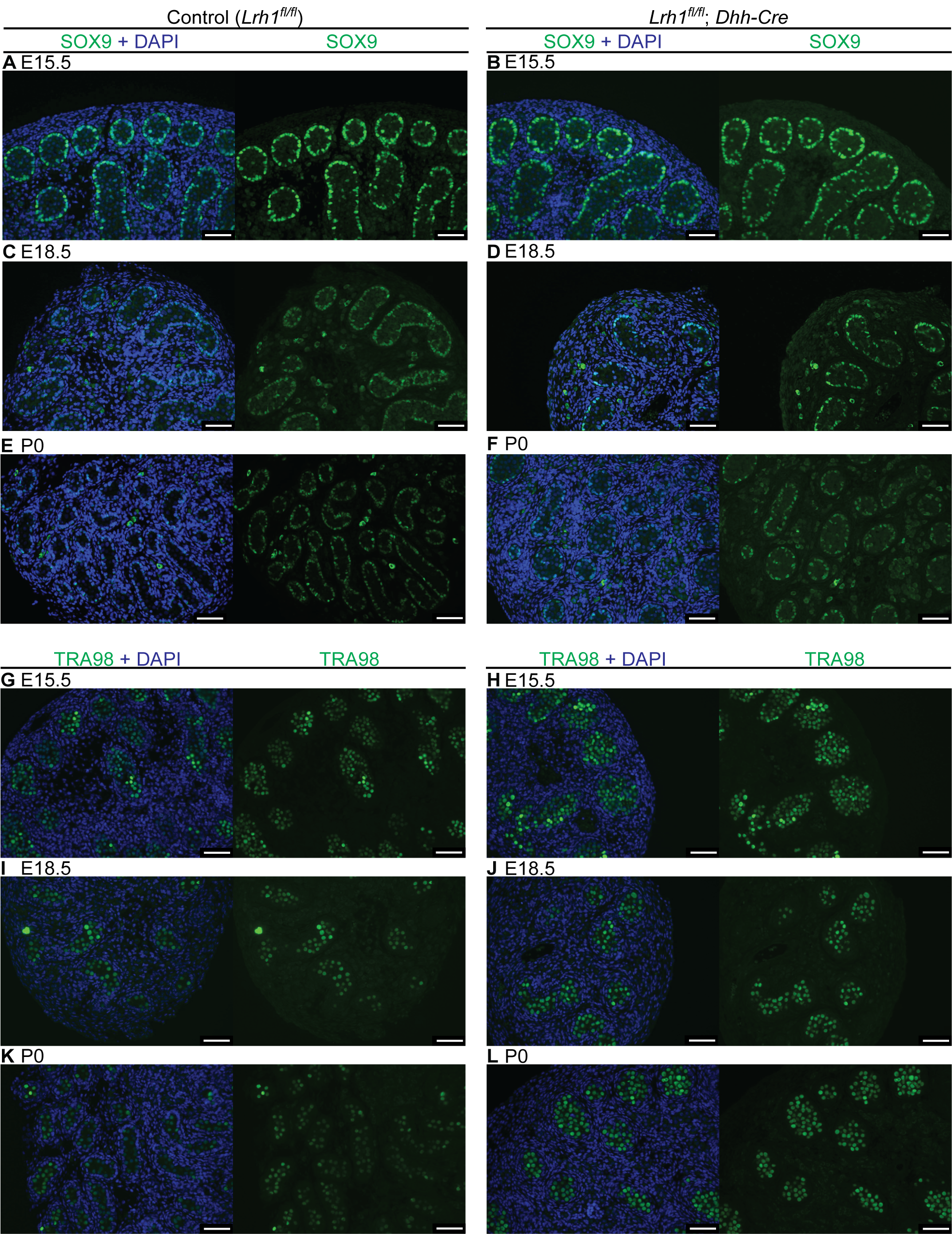

Supplement: S3 Fig — (A-F) IF staining of testis sections for Sertoli marker SOX9 (green) and DAPI (blue) comparing controls lacking Cre and Dhh-Cre deleted Lrh1 mutants. (A,B) E15.5, (C,D) E18.5, (E,F). P0 (G-L) IF staining of testis sections for germ cell marker TRA98 (green) and DAPI (blue) comparing controls lacking Cre and Dhh-Cre deleted Lrh1 mutants. (G,H) E15.5, (I,J) E18.5, (K,L) P0. Scale bars: 50 um. (TIF) [file pgen.1010088.s003.tif]

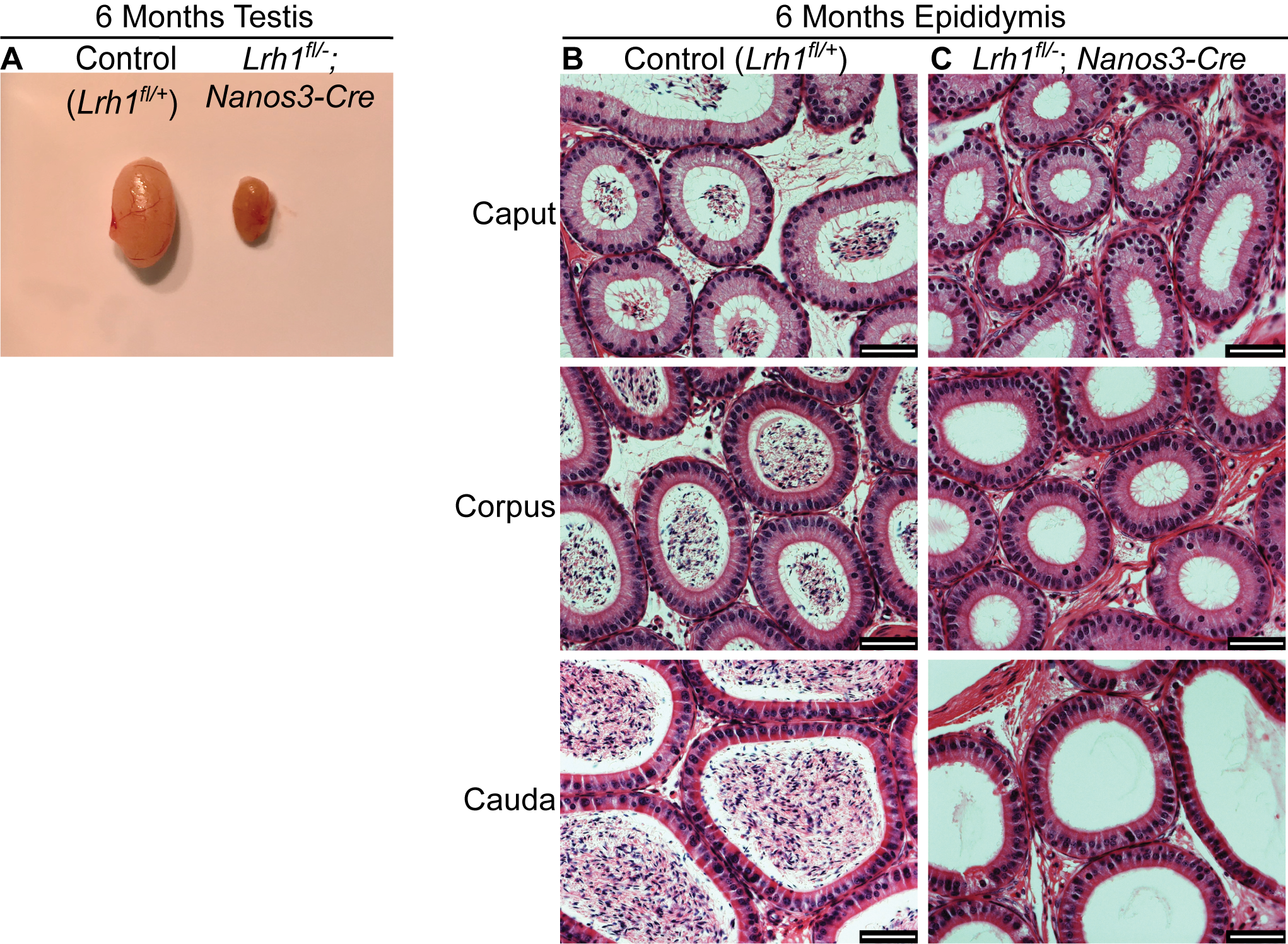

Supplement: S4 Fig — (A) Intact testes from control and Nanos3-Cre deleted germ cell conditional mutant at 6 months. (B,C) H&E stained sections of epididymi from control and germ cell conditional mutants. Scale bars: 50 um. (TIF) [file pgen.1010088.s004.tif]

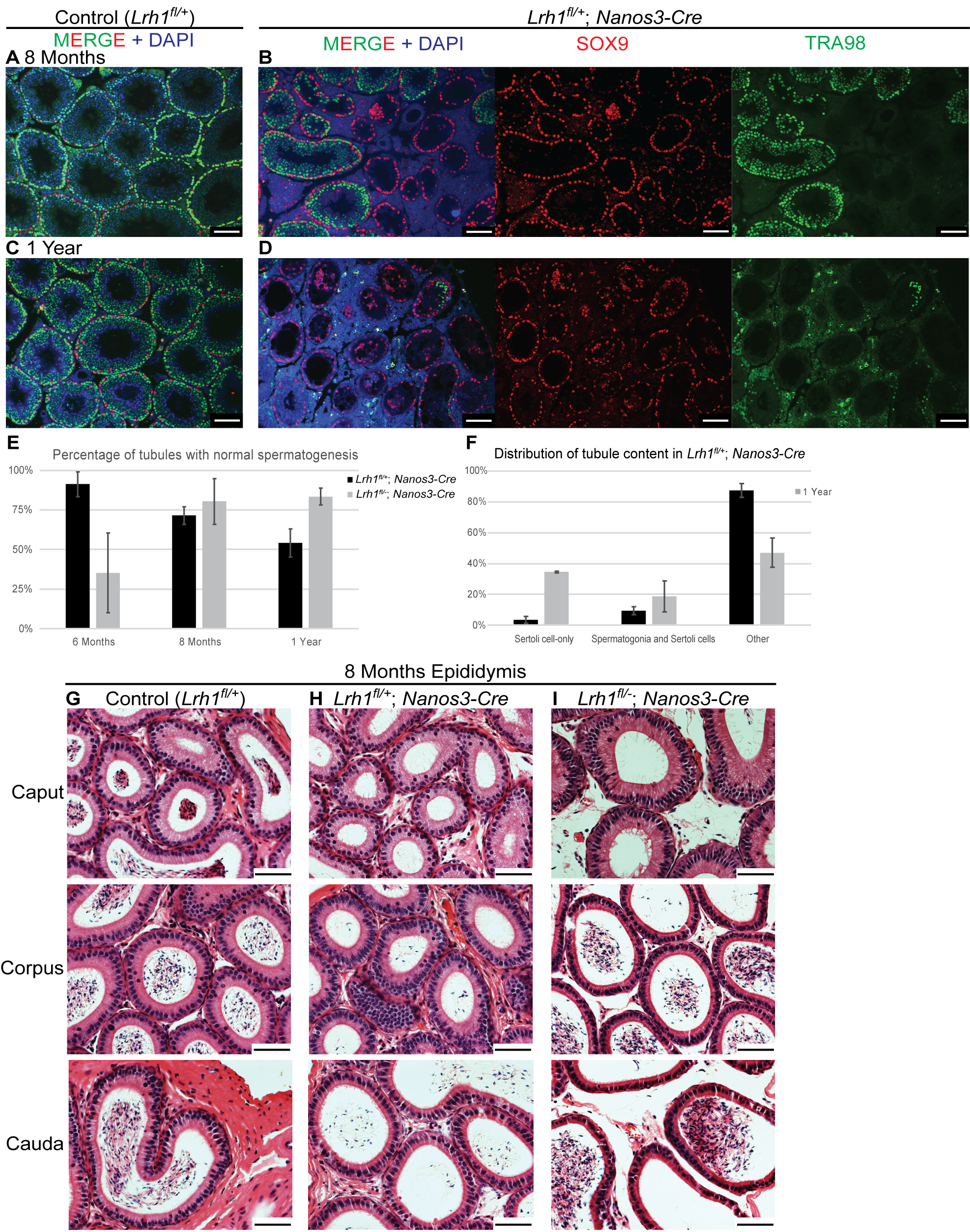

Supplement: S5 Fig — (A-D) IF of testis sections stained for SOX9 (red), TRA98 (green) and DAPI (blue) showing presence of both Sertoli-only and spermatogenic tubules at 8 months (A,B) and mainly Sertoli-only tubules at 1 year (C,D). (E,F) Plots comparing percentage of seminiferous tubules with normal spermatogenesis in Lrh1 heterozygous and homozygous germ cell conditional mutant testes (E) and distribution of tubule germ cell content (F) from 6 months to one year. (“Other” indicates mix of spermatogonia and advanced germ cells.) Error bars: SEM. (G-I) H&E stained sections of epididymi from control and germ cell conditional Lrh1 mutants showing abundant epididymal sperm in homozygous mutant (I) but not in heterozygous mutant (H) at 8 months. White scale bars: 100 um. Black scale bars: 50 um. (TIF) [file pgen.1010088.s005.tif]

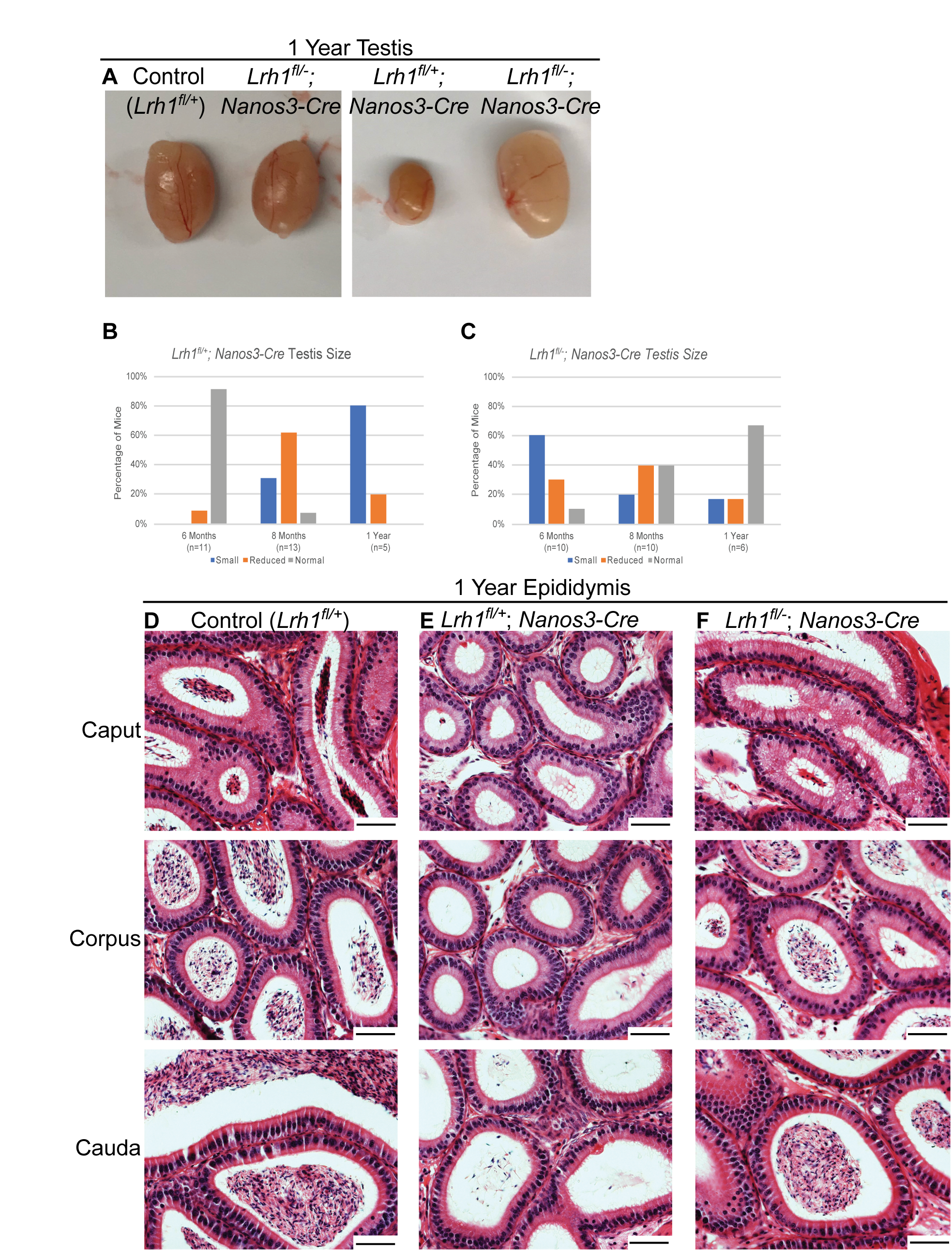

Supplement: S6 Fig — (A) Lrh1 homozygous Nanos3-Cre deleted germ cell mutant testes are normal size and heterozygotes are severely reduced in size relative to controls at one year. (B-C) Plots showing observed testis size of collected heterozygous and homozygous mutant mice at 6 months, 8 months, and 1 year. (D-F) H&E stained sections of epididymi from control and germ cell conditional mutants showing abundant epididymal sperm in homozygous mutant (F) but not in heterozygous mutant (E) at 1 year. Scale bars: 50 um. (TIF) [file pgen.1010088.s006.tif]
